# Aetiology of community-acquired neonatal sepsis in low and middle income countries

**Published:** 2011-12

**Authors:** Donald Waters, Issrah Jawad, Aziez Ahmad, Ivana Lukšić, Harish Nair, Lina Zgaga, Evropi Theodoratou, Igor Rudan, Anita K. M. Zaidi, Harry Campbell

**Affiliations:** 1 Centre for Population Health Sciences and Global Health Academy, The University of Edinburgh, Scotland, UK; 2 Department of Paediatrics and Child Health, Aga Khan University, Karachi, Pakistan; 3 Department of Microbiology, Dubrava University Hospital, Zagreb, Croatia; * Joint senior authorship

## Abstract

**Background:**

99% of the approximate 1 million annual neonatal deaths from life-threatening invasive bacterial infections occur in developing countries, at least 50% of which are from home births or community settings. Data concerning aetiology of sepsis in these settings are necessary to inform targeted therapy and devise management guidelines. This review describes and analyses the bacterial aetiology of community-acquired neonatal sepsis in developing countries.

**Methods:**

A search of Medline, Embase, Global Health and Web of Knowledge, limited to post-1980, found 27 relevant studies. Data on aetiology were extracted, tabulated and analysed along with data on incidence, risk factors, case fatality rates and antimicrobial sensitivity.

**Results:**

The most prevalent pathogens overall were *Staphylococcus aureus* (14.9%), *Escherichia coli* (12.2%), and *Klebsiella* species (11.6%). However, variations were observed both between global regions and age-of-onset categories. *Staphylococcus aureus* and *Streptococcus pneumoniae* were most prevalent in Africa, while *Klebsiella* was highly prevalent in South-East Asia. A notably higher prevalence of Group B *Streptococcus* was present in neonates aged 7 days or less. The highest case fatality rates were recorded in South-East Asia. *Klebsiella* species showed highest antimicrobial resistance.

**Conclusion:**

Data on community-acquired neonatal sepsis in developing countries are limited. Future research should focus on areas of high disease burden with relative paucity of data. Research into maternal and neonatal vaccination strategies and improved diagnostics is also needed. All of this could contribute to the formulation of community-based care packages, the implementation of which has significant potential to lower overall neonatal mortality and hence advance progress towards the attainment of Millennium Development Goal 4.

Approximately 1 million deaths a year occurring in the neonatal period (0-28 days) are caused by infection, accounting for over 25% of global neonatal deaths and 10% of all mortality in infants under the age of 5 (1); 99% of these deaths occur in developing countries (2). Neonatal sepsis is classically defined as the presence of symptoms of sepsis in the neonatal period combined with bacteriological isolation of an infectious agent from blood or cerebrospinal fluid (CSF) (3). It is classified as ‘early-onset’ if it occurs within the first 7 days of life and as ‘late-onset’ if it occurs after this time. Typically, early-onset sepsis is considered maternally-acquired, usually from the maternal genital tract, and late-onset sepsis is generally regarded to originate from the caregiving environment – either a health care or community setting. Consequently early- and late-onset sepses are also associated with different distributions of pathogens (4).

The majority of babies in developing countries are born at home and at least a half of neonatal deaths occur in home births (5). Reasons for this include poor health system coverage or provision and limited or no access to referral facilities (6). There is significant evidence that in rural areas neonates often do not receive required health care and that this is associated with an increase in mortality (7). However, other reasons for the high prevalence of home births can be suggested, such as financial constraints. This is because even when health care services are available, and of respectable quality, they may still remain beyond the financial means of many (8). There are also potential sociocultural issues related to the rejection of health care services for newborns, because research has demonstrated a high prevalence of refusal of hospital referrals by their families and highlighted the need for education programmes on appropriate care seeking (9).

The predominance of home births in developing countries is not reflected in related research as this mainly provides data on neonatal sepsis in hospitals, a large percentage of which is nosocomial (10). There are several potential reasons behind the lack of aetiological data on neonatal sepsis acquired in the community, including lack of sufficient laboratory facilities in rural areas and also potentially low levels of care seeking, resulting in much unreported morbidity and mortality. This may particularly be true for the cases that occur in the areas without access to health care, or in areas with poorly developed care-seeking behaviour (4). This review is concerned specifically with the bacterial aetiology of life-threatening, community-acquired neonatal sepsis (CANS).

## Pathogenesis and risk factors

Due to their immature immune systems and incompletely developed skin barriers, neonates are more susceptible to infection (11). In developing countries, the likelihood of infection is increased due to other additional risk factors. Unsafe birthing practices are common, with only 35% of births in some of the least developed countries being attended by a skilled birth attendant (12), often resulting in unhygienic practices such as delivery onto a unsterile floor, unsterile cord cutting and potentially unsafe cultural customs such as spreading dung on the newborn’s umbilicus (10). Other predisposing risk factors for infection in neonates include low birth-weight, prematurity, prolonged rupture of membranes and a long delivery period (3).

Health education can also be a problem with early detection of CANS in developing countries often being low, potentially due to mothers failing to notice important symptoms and seek health care. The role of women in some societies is also an issue, with woman having a low social standing and a lack of autonomy resulting in delays in, or absence of, care seeking for infant’s health, poorer sanitation and a decrease in access to health care facilities (10).

## Management

Management of neonatal sepsis in a hospital setting is commonly through parenteral antibiotic therapy and supportive care, which has shown positive impacts (10). However, it is important to note that most neonates in developing countries do not receive this therapy because they do not have access to the necessary health services, or their parents do not seek care. A recent review showed a significant reduction in mortality from CANS as a result of introducing perinatal care packages including injectable antibiotics to the community (6). Research shows that the aetiology of neonatal sepsis is continually evolving, and therefore continuing updating of aetiological data are necessary to inform appropriately targeted therapy (10). A previous review of CANS showed a predominance of gram-negative organisms over gram-positive, with the main causative pathogens being *Klebsiella* species, *Escherichia coli* and *Staphylococcus aureus* (4). The focus of this review will be to provide updated data on the aetiology of CANS globally.

## Prevention

In addition to efforts to improve diagnosis and treatment of CANS, efforts to prevent this life-threatening illness are also important to consider. A review of possible preventative interventions for improving neonatal health highlighted a need for universal provision of antenatal care for mothers in developing countries as a means of decreasing mortality from neonatal sepsis (13). This involves educating mothers about hygienic birth practice, promoting breast feeding and also detecting and treating important maternal risk factors for neonatal sepsis, such as asymptomatic bacteriuria (13). Another important potential way to prevent neonatal sepsis is to train and provide adequate numbers of skilled birth attendants in the community (10).

Possibly one of the most important preventative interventions after birth is early and exclusive breastfeeding. Breast milk contains important immunological factors, some of which have the potential to inhibit causative pathogens of neonatal sepsis (11). This is a major issue, as recent research has shown that only 37% of infants younger than six months of age in developing countries are exclusively breastfed (14). Consequently, promotion of breastfeeding in community settings is the subject of an extensive World Health Organization (WHO) strategy document (15).

## International responses to neonatal sepsis

Neonatal sepsis is an important issue internationally, especially with relation to the United Nations Millennium Development Goals. Without a reduction in newborn deaths, of which sepsis is a major cause, the fourth goal of reducing mortality in children under five by two-thirds cannot be achieved (2). There is therefore a need to investigate prevention, diagnosis and treatment strategies and their potential for implementation or improvement globally (8).

Founded in 1992 by the WHO and the United Nations Children's Fund (UNICEF), the Integrated Management of Childhood Illness initiative (IMCI) is an integrated approach to improving child health globally, which provides guidelines including curative and preventative elements in both health care and community settings. Rather than an individual disease-specific approach, IMCI has a wide and integrated strategy, aiming at addressing the varied risk factors for childhood illness (16). This approach is highly relevant in the case of neonatal sepsis as the disease has many risk factors and can be both health care- and community-acquired. In many settings where CANS is prevalent, high-quality diagnostic facilities are not widely available and the determination of commonly observed clinical signs provided by IMCI guidelines could be key in increasing diagnosis of neonatal sepsis and improving health outcomes (10).

Emerging antibiotic resistance is also important to consider in relation to CANS. A recent review in this area concluded that data concerning antibiotic resistance in CANS are very limited, but nevertheless highlighted potential cause for concern resulting from studies showing emerging resistance in *Klebsiella* species and *E coli* although levels of resistance were noted to be lower than in hospital settings (17). Emerging antibiotic resistance is a major international concern (18) and the information about the aetiological spectrum of CANS and the prevalence of antibiotic resistance among major causal pathogens are important to build a broader understanding of this important public health issue.

## Aims of this study

The aims of this study were to provide information on the bacterial aetiology of CANS in developing countries and to discuss the implications of the information generated for future research and international child health policy in this field. The specific objectives of this systematic literature review were to determine the bacterial aetiology of CANS in developing countries through systematic literature review, to investigate aetiological variations between global regions and different ages-of-onset and to explore potential suggestions from information presented for future policy and research.

## METHODS

A review of published literature was undertaken using the electronic databases Medline, Embase, Global Health and Web of Knowledge. The search involved combinations of Medical Subject Headings (MeSH) and keywords in conjunction with a search for each individual developing country. These were defined as low- or middle-income countries from World Bank classifications (19). Search terms used for Medline and Embase are shown in **Table 1**. Terms for other databases were slightly modified where necessary, to fit the search terms offered in the respective databases. Final searches on all databases were undertaken on 30 January 2011. Searches were supplemented by screening reference lists of selected papers and including literature discovered that corresponded with inclusion criteria.

**Table 1:** Search terms for Medline/Embase

1.	Developing Countries/ or Algeria/ or Egypt/ or Libya/ or Morocco/ or Tunisia/ or Cameroon/ or Central African Republic/ or Chad/ or Congo/ or “Democratic Republic of the Congo”/ or Gabon/ or Burundi/ or Djibouti/ or Eritrea/ or Ethiopia/ or Kenya/ or Rwanda/ or Somalia/ or Sudan/ or Tanzania/ or Uganda/ or Angola/ or Botswana/ or Lesotho/ or Malawi/ or Mozambique/ or Namibia/ or South Africa/ or Swaziland/ or Zambia/ or Zimbabwe/ or Benin/ or Burkina Faso/ or Cape Verde/ or Cote d'Ivoire/ or Gambia/ or Ghana/ or Guinea/ or Guinea-Bissau/ or Liberia/ or mail/ or Mauritania/ or Niger/ or Nigeria/ or Senegal/ or Sierra Leone/ or Togo/ or “Antigua and Barbuda”/ or Cuba/ or Dominica/ or Dominican Republic/ or Grenada/ or Haiti/ or Jamaica/ or “Saint Kitts and Nevis”/ or Saint Lucia/ or “Saint Vincent and the Grenadines”/ or Belize/ or Costa Rica/ or El Salvador/ or Guatemala/ or Honduras/ or Nicaragua/ or Panama/ or Mexico/ or Argentina/ or Bolivia/ or Brazil/ or Chile/ or Colombia/ or Ecuador/ or Guyana/ or Paraguay/ or Peru/ or Suriname/ or Uruguay/ or Venezuela/ or Antarctic Regions/ or Arctic Regions/ or Kazakhstan/ or Kyrgyzstan/ or Turkmenistan/ or Uzbekistan/ or Borneo/ or Cambodia/ or East Timor/ or Indonesia/ or Laos/ or Malaysia/ or Mekong Valley/ or Myanmar/ or Philippines/ or Thailand/ or Vietnam/ or Bangladesh/ or Bhutan/ or India/ or Sikkim/ or Afghanistan/ or Iran/ or Iraq/ or Jordan/ or Lebanon/ or Syria/ or Turkey/ or Yemen/ or Nepal/ or Pakistan/ or Sri Lanka/ or China/ or Hong Kong/ or Macau/ or Tibet/ or Korea/ or “Democratic People's Republic of Korea”/ or Mongolia/ or Taiwan/ or Albania/ or Lithuania/ or Bosnia-Herzegovina/ or Bulgaria/ or “Republic of Belarus”/ or “Macedonia (republic)”/ or Moldova/ or Montenegro/ or Russia/ or Bashkiria/ or Dagestan/ or Moscow/ or Siberia/ or Serbia/ or Ukraine/ or Yugoslavia/ or Armenia/ or Azerbaijan/ or “Georgia (republic)”/ or Melanesia/ or Fiji/ or Papua New Guinea/ or Vanuatu/ or Micronesia/ or Palau/ or Polynesia/ or Samoa/ or “Independent State of Samoa”/ or Tonga/ or Comoros/ or Madagascar/ or Mauritius/ or Seychelles/ or Solomon Islands.mp. or Marshall Islands.mp. or (Sao Tome and Principe).mp. or Maldives.mp. or Tuvalu.mp. or (West Bank and Gaza).mp. or American Samoa/ or Romania/
2.	exp Infant, Newborn/ or (newborn* or neonat*).tw.
3.	exp sepsis/ or exp infection/ or (infection* or pathogen* or organism* or bacter* or etiology).tw.
4.	Limit 3 to “etiology (sensitivity)” [Limit not valid in Embase; records were retained]
5.	(neonat* adj3 sepsis).tw.
6.	2 and 4
7.	5 or 6
8.	1 and 7

### Inclusion and exclusion criteria

Although it started with no time limits, the review was eventually restricted to literature published after 1980, to limit the number of studies and to present current aetiological data. No restrictions were used concerning publication type or language of publication. All life-threatening invasive bacterial infections (bacteraemia, pneumonia and meningitis) affecting infants of 0-90 days of age were included. Studies reporting viral or fungal infection, nosocomial infection, congenital infection or other infections such as *ophthalmia neonatorum*, malaria, tetanus or tuberculosis were excluded. All studies reporting CANS were included, along with studies where the setting or infection type reported suggested CANS. A certain number of studies were found where it was thought that CANS was indicated; however, the study data were deemed inconclusive to justify this assumption with a sufficient degree of certainty. These studies were included for data extraction, but not for the final data analysis. Isolated organisms from both blood and CSF were included.

Studies reporting less than 50 cases were excluded to increase the potential to generalise from results and prevent large deviations in suggested prevalence due to small sample sizes and chance effects. Studies reporting the incidence/prevalence of only one organism were also excluded, for the same reason of likely over-estimation and the effects of chance. Review articles were also excluded, because primary data were the focus of the review. These were, however, used as helpful sources of reference.

### Data extraction

Data were extracted from all selected studies and compiled in Microsoft Excel spreadsheets. An overall table of study characteristics was formed (**Supplementary Table 1**)(Supplementary Table 1) and individual tables for each study were compiled, charting quantities of organisms isolated from blood-culture proven CANS (**Supplementary Table 2**)(Supplementary Table 2) (20-46). This data was further split into ≤7 days of life, 7-59 days of life and 60-90 days of life based on the neonatal age at isolation of organism, henceforth described as age-of-onset (**Supplementary Table 3**)(Supplementary Table 3). The first category corresponds to early-onset sepsis as described in the introduction. The second category corresponds to late-onset sepsis but was expanded to fit the WHO definition of a ‘young infant’ (7-59 days). The third category includes any data after this period up to 90 days. For several studies data were not reported in the categories described above and so it was necessary to redistribute so as to standardise for analysis. Some studies reported overlapping aetiological data for CSF and blood isolates (i.e. more than one isolate for an individual patient), in these studies it was decided to only extract data on blood isolates to avoid distortion of the results.

It was decided through the study of primary data descriptions and previous reviews not to extract data for certain organisms, including *Myma polymorpha* and *Micrococci* sp. as they were deemed to be contaminants. Although previous studies have excluded altogether data specifying infection from coagulase-negative *Staphylococci*, which are known to be common opportunistic pathogens in hospital-acquired infections, but are seen as likely to be contaminants in CANS (4,47), it was decided to extract data for these organisms but exclude them from summary tables.

### Data analysis

**Figure 1:**
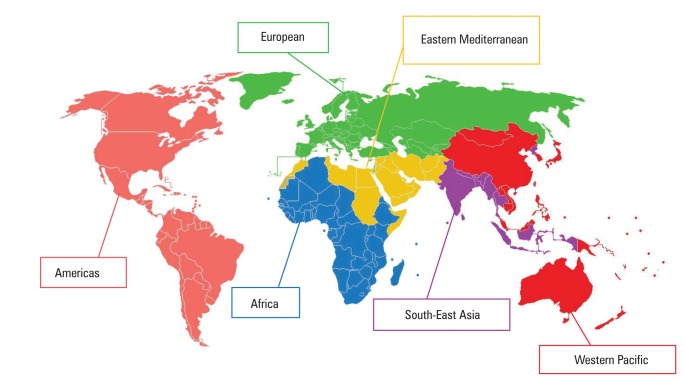
WHO Regions (adapted from Wikimedia Commons; http://commons.wikimedia.org/wiki/File:World_Health_Organisation_regional_offices.PNG).

For the purpose of analysis, data tables from studies were separated into 6 WHO global regions as illustrated below (**Figure 1**). Summary tables using the aetiological categories above were assembled for each region and relative percentages of each organism calculated (**Supplementary Table 4**)(Supplementary Table 4). Tables for each region were then compiled, detailing only potentially pathogenic organisms so as to gain clearer insight into aetiology. Decisions on pathogenicity were based on those of previous reviews and also the information from other published literature (2,4,48). The ‘other/ unspecified’ category for Gram-positives and Gram-negatives was removed from potential pathogen tables along with the Non-stated/Undetermined category.

Regional relative percentages for potential pathogens were calculated along with 95% confidence intervals and these were combined through meta-analysis to counteract issues with data bias and create an ‘All Regions’ category (**Supplementary Table 5**)(Supplementary Table 5). Either the fixed effect model (Mantel-Haenszel method) or in cases of heterogeneity the random effect model (DerSimonian-Laird method) were used (49). Between-study heterogeneity was quantified by calculating the Q statistic with a p-value less or equal than 0.05 being the threshold (49). The meta-analysis results were unstable for several pathogens due to the small quantity of data, therefore it was decided for the purpose of analysis to use median and inter-quartile (IQ) range data from regional percentages for all potential pathogens and meta-analytical data of regional percentages for the cumulative ‘Potentially pathogenic Gram-positives/Negatives’ columns.

As 30% of studies reported aetiological data using just numbers of positive isolates for each organism as a denominator rather than the number of patients with positive isolates for each organism, some studies reported more isolates than the number of patients in their sample. Therefore it was deemed necessary to split aetiological data into that based on patients and that based on isolates so as to determine and analyse any differences. Other data on incidence, case fatality rates, risk factors for CANS and antimicrobial susceptibility patterns were extracted where available and analysed.

### Quality control

To ensure quality control, another reviewer undertook an independent second data extraction of a certain proportion of this review’s selected studies, totaling 520 data points; 100% of these were the same and therefore it was concluded that the standard of data reliability was likely to be high.

## RESULTS

**Figure 2:**
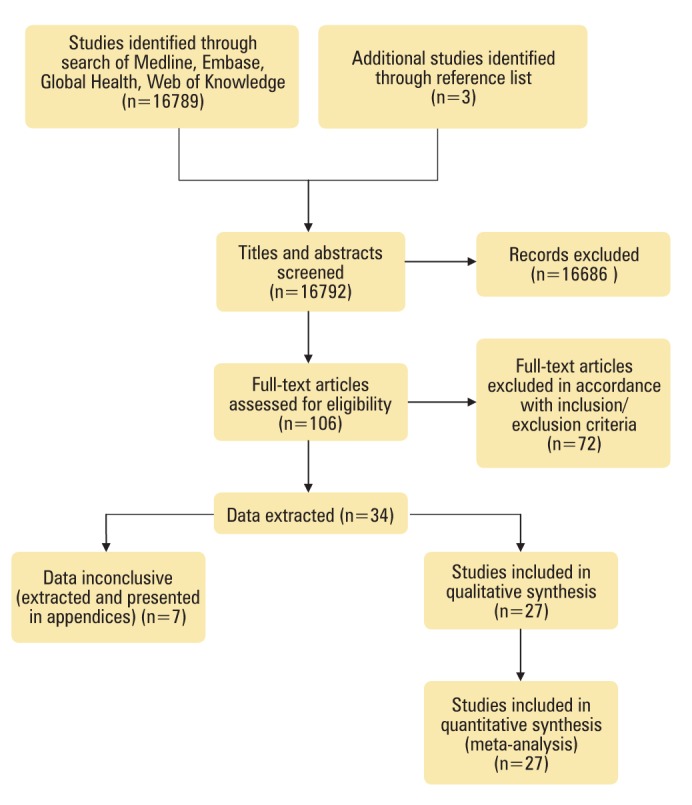
An overview of literature search results.

The literature search returned 16 789 studies whose titles and abstracts were reviewed for relevance. 103 were selected for full text examination, however only 100 papers were sourced in full-text versions. Of these 31 were selected for inclusion in the review. In addition, 3 studies were found from other studies’ reference lists and selected for inclusion, resulting in a total of 34 studies included in the review. Of these, 27 studies were deemed to be reporting data concerning CANS and 7 were considered to be less conclusive, possibly reporting neonatal sepsis acquired from another source (**Supplementary Table 6**)(Supplementary Table 6) (50-56). To avoid potential compromising of final results it was decided to extract data from these 7 papers however exclude them from overall analyses. The literature search process is outlined in **Figure 2**.

Characteristics of the studies that were retained after the process of literature search as they met the minimum quality criteria are shown in **Table 2**. A full version of this table can be found in **Supplementary Table 1**(Supplementary Table 1). Of the 27 studies, only 2 presented community surveillance data. Another 20 either presented CANS-specific or disaggregated non-nosocomial data and the remaining 5 did not explicitly report CANS aetiology but were deemed suitable for inclusion due to the infection type or study setting reported. Four studies were the primary data sources for a WHO Young Infants Study Group Multicenter Study (47), which presented overall data. However, in our study we treated each site as an independent data point, and we analysed the information from each study individually.

**Table 2:** Study characteristics

Characteristic		No. studies
WHO Region	Africa	11
	Americas	1
	Europe	2
	Eastern Mediterranean	1
	South-East Asia	7
	Western Pacific	5
Length of study	<1 Year	3
	1 - 2 Years	9
	3 - 4 Years	8
	≥5 Years	5
	Not reported	2
Number of studies active in particular time periods	1980-1985	2
	1986-1990	5
	1991-1995	13
	1996-2000	11
	2001-2005	10
	2006-2010	2
	Not reported	2
Culture category	Blood	16
	CSF	5
	Blood and/or CSF*	5
	Urine/Other	2
Study denominator	Isolates	8
	Patients	19
Number of positive isolates	0-25	8
	26-50	10
	51-75	5
	76-100	1
	101-200	1
	>200	2
Number of potentially pathogenic positive isolates	0-25	11
	26-50	10
	51-75	3
	76-100	1
	101-200	1
	>200	1

CSF – cerebrospinal fluid

*One study also used antigen detection.

### Aetiological data

Individual aetiological data from each study are presented in **Supplementary Table 2**(Supplementary Table 2). For the purpose of analyses, aetiological data were split by WHO regions, and then by age-of-onset. **Table 3** is a summary table that contains data for all isolated organisms by region. **Table 4** contains data for all potential pathogens isolated by region, both for age-of-onset categories ≤7 days of life and 8-59 days. Data for the 60-90 days of life category were excluded from these tables to fit with the WHO ‘young infant’ criteria. Full tables including this data are presented in **Supplementary Table 4**(Supplementary Table 4).

**Table 3:** Distribution of all microorganisms isolated by region

	Africa		Americas		Eastern Mediterranean		Europe		South-East Asia		Western Pacific		All Regions			
Microorganism isolated	N	% (95%CI)	N	% (95%CI)	N	% (95%CI)	N	% (95% CI)	N	% (95% CI)	N	% (95% CI)	N	% (95% CI)	Median	IQ range
*Staphylococcus aureus*	85	14.6 (11.7 - 17.4)	0	0.0 (-)	0	0.0 (-)	28	26.4 (19.0 - 35.6)	20	10.0 (6.6 - 14.9)	168	15.2 (13.2 - 17.5)	301	14.6 (13.1 - 16.2)	12.3	12.6
Group A *Streptococci/ Streptococcus pyogenes*	40	6.9 (4.8 - 8.9)	0	0.0 (-)	0	0.0 (-)	0	0.0 (-)	2	1.0 (0.3 - 3.6)	12	1.1 (0.6 - 1.9)	54	2.6 (2.0 - 3.4)	0.5	1.1
Group B *Streptococci*	40	6.9 (4.8 - 8.9)	0	0.0 (-)	0	0.0 (-)	1	0.9 (0.2 - 5.2)	6	3.0 (1.4 - 6.4)	3	0.3 (0.1 - 0.8)	50	2.4 (1.8 - 3.2)	0.6	2.4
Group D *Streptococci/ Enterococcus*	4	0.7 (0.0 - 1.4)	0	0.0 (-)	2	4.8 (1.3- 15.8)	9	8.5 (4.5 - 15.4)	0	0.0 (-)	2	0.2 (0.0 - 0.7)	17	0.8 (0.5 - 1.3)	0.4	3.7
*Streptococcus pneumoniae*	81	13.9 (11.1 - 16.7)	1	3.0 (0.5 - 15.3)	3	7.1 (2.5 - 19.0)	0	0.0 (-)	7	3.5 (1.7 - 7.0)	14	1.3 (0.8 - 2.1)	106	5.1 (4.3 - 6.2)	3.3	4.5
Other/unspecified *Streptococcus* species	19	3.3 (2.1 - 5.0)	0	0.0 (-)	10	23.8 (13.5 - 38.5)	0	0.0 (-)	1	0.5 (0.1 - 2.8)	44	4.0 (3.0 - 5.3)	74	3.6 (2.9 - 4.5)	1.9	3.7
Other/ unspecified Gram-positives*	1	0.2 (0.0 - 1.0)	0	0.0 (-)	0	0.0 (-)	0	0.0 (-)	1	0.5 (0.1 - 2.8)	110	10.0 (8.3 - 11.9)	112	5.4 (4.5 - 6.5)	0.1	0.4
**Total Gram-positives**	**270**	**46.3 (42.3 - 50.4)**	**1**	**3**	**15**	**35.7 (23.0 - 50.8)**	**38**	**35.8 (27.4 - 45.3)**	**37**	**18.5 (13.7 - 24.5)**	**353**	**32.0 (29.3 - 34.8)**	**714**	**34.6 (32.5 - 36.6)**	**33.9**	13.9
*Klebsiella pneumoniae*	22	3.8 (2.5 - 5.6)	0	0.0 (-)	2	4.8 (1.3 - 15.8)	20	18.9 (12.6 - 27.4)	67	33.5 (27.3 - 40.3)	143	13.0 (11.1 - 15.1)	254	12.3 (10.9 - 13.8)	8.9	13.4
Other/unspecified *Klebsiella* species	20	3.4 (2.2 - 5.2)	0	0.0 (-)	0	0.0 (-)	0	0.0 (-)	5	2.5 (1.1 - 5.7)	0	0.0 (-)	25	1.2 (0.8 - 1.8)	0	1.9
*Escherichia coli*	64	11.0 (8.7 - 13.8)	0	0.0 (-)	3	7.1 (2.5 - 9.0)	38	35.8 (27.4 - 45.3)	18	9.0 (5.8 - 13.8)	243	22.1 (19.7 - 24.6)	366	17.7 (16.1 - 19.4)	10	11.7
*Pseudomonas* species	22	3.8 (2.5 - 5.6)	0	0.0 (-)	4	9.5 (3.8 - 22.1)	4	3.8 (1.5 - 9.3)	18	9.0 (5.8 - 13.8)	140	12.7 (10.9 - 14.8)	188	9.1 (7.9 - 10.4)	6.4	5.6
*Enterobacter* species	5	0.9 (0.4 - 2.0)	0	0.0 (-)	4	9.5 (3.8 - 22.1)	1	0.9 (0.2 - 5.2)	3	1.5 (0.5 - 4.3)	56	5.1 (4.0 - 6.5)	69	3.3 (2.6 - 4.2)	1.2	3.3
*Serratia* species	0	0.0 (-)	0	0.0 (-)	0	0.0 (-)	1	0.9 (0.2 - 5.2)	0	0.0 (-)	39	3.5 (2.6 - 4.8)	40	1.9 (1.4 - 2.6)	0	0.7
*Proteus* species	9	1.5 (0.8 - 3.0)	0	0.0 (-)	0	0.0 (-)	1	0.9 (0.2 - 5.2)	0	0.0 (-)	3	0.3 (0.1 - 0.8)	13	0.6 (0.4 - 1.1)	0.1	0.8
*Salmonella* species	17	2.9 (1.8 - 4.6)	0	0.0 (-)	0	0.0 (-)	0	0.0 (-)	3	1.5 (0.5 -4.3)	7	0.6 (0.3 - 1.3)	27	1.3 (0.9 - 1.9)	0.3	1.3
*Haemophilus influenzae*	25	4.3 (2.9 - 6.3)	3	9.1 (3.1 - 23.6)	1	2.4 (0.4 - 12.3)	0	0.0 (-)	1	0.5 (0.1 - 2.8)	2	0.2 (0.0 - 0.7)	32	1.5 (1.1 - 2.2)	1.4	3.6
*Neisseria meningitidis*	11	1.9 (1.1 - 3.3)	2	6.1 (1.7 - 19.6)	0	0.0 (-)	0	0.0 (-)	0	0.0 (-)	0	0.0 (-)	13	0.6 (0.4 - 1.1)	0	1.4
*Acinetobacter* species	26	4.5 (3.1 - 6.5)	0	0.0 (-)	2	4.8 (1.3 - 15.8)	0	0.0 (-)	9	4.5 (2.4 - 8.3)	95	8.6 (7.1 - 10.4)	132	6.4 (5.4 - 7.5)	4.5	3.6
Other/unspecified Gram-negatives**	59	10.1 (7.9 - 12.8)	0	0.0 (-)	11	26.2 (15.3 - 41.1)	0	0.0 (-)	11	5.5 (3.1 - 9.6)	20	1.8 (1.2 - 2.8)	101	4.9 (4.0 - 5.9)	3.7	8.5
**Total Gram-negatives**	**280**	**48.0 (44.0 - 52.1)**	**5**	**15.2 (6.7 - 31.0)**	**27**	**64.3 (49.1 - 77.0)**	**65**	**61.3 (51.8 - 70.0)**	**135**	**67.5 (60.7 - 73.6)**	**748**	**67.9 (65.1 - 70.6)**	**1260**	**61.0 (58.9 - 63.1)**	**62.8**	15.3
**Non-stated/Undetermined**	33	5.7 (4.1 -7.8)	27	81.8 (65.6 - 91.4)	0	0.0 (-)	3	2.8 (1.0 - 8.0)	28	14.0 (9.9 - 19.5)	1	0.1 (0.0 - 0.5)	92	4.5 (3.6 - 5.4)	4.2	11.1
**Total**	**583**	**100.0 (n/a)**	**33**	**100.0 (n/a)**	**42**	**100.0 (n/a)**	**106**	**100.0 (n/a)**	**200**	**100.0 (n/a)**	**1102**	**100.0 (n/a)**	**2066**	**100.0 (n/a)**		

95% CI – 95% confidence interval, IQ range – interquartile range

* Includes data *for Aerococcus* sp, *Bacillus* sp. and others.

**Includes data for *Citrobacter* sp, *Moraxella* sp, *Shigella* sp, *Aeromonas* sp. and others. Data was also extracted for coagulase-negative *Staphylococci*: Africa – 28 isolates, Americas – 0 isolates, Eastern Mediterranean – 0 isolates, Europe – 9 isolates, South-East Asia – 35 isolates, Western Pacific – 811 isolates.

**Table 4:** Distribution of potential pathogens isolated by region

	Africa		Americas		Eastern Mediterranean		Europe		South-East Asia		Western Pacific		All Regions				
Potential pathogen isolated	N	% (95% CI)	N	% (95% CI)	N	% (95% CI)	N	% (95% CI)	N	% (95% CI)	N	% (95% CI)	N	% (95% CI)	Median % (IQ range)	Meta-Analysis % (95% CI)	P-value for heterogeneity
*Staphylococcus aureus*	85	17.3 (14.3 - 21.0)	0	0.0 (-)	0	0.0 (-)	28	27.2 (19.5 - 36.5)	20	12.5 (8.2 -18.5)	168	17.3 (15.1 - 19.8)	301	17.1 (15.4 - 18.9)	14.9 (14.2)	17.4 (13.9 - 20.9)	0.036
Group A *Streptococci/ Streptococcus pyogenes*	40	8.2 (6.1 - 10.9)	0	0.0 (-)	0	0.0 (-)	0	0.0 (-)	2	1.3 (0.3 - 4.4)	12	1.2 (0.7 - 2.1)	54	3.1 (2.4 - 4.0)	0.6 (1.2)	3.4 (0.0 - 7.2)	<0.0005
Group B *Streptococci*	40	8.2 (6.1 - 10.9)	0	0.0 (-)	0	0.0 (-)	1	1.0 (0.2 - 5.3)	6	3.8 (1.7 - 7.9)	3	0.3 (0.1 - 0.9)	50	2.8 (2.1 - 3.7)	0.6 (3)	3.2 (0.0 - 7.0)	<0.0005
Group D *Streptococci/ Enterococcus*	4	0.8 (0.3 - 2.1)	0	0.0 (-)	2	6.5 (1.8 - 20.7)	9	8.7 (4.7 - 15.8)	0	0.0 (-)	2	0.2 (0.1 - 0.7)	17	1.0 (0.6 - 1.5)	0.5 (5)	1.1 (0.0 - 2.5)	0.007
*Streptococcus pneumoniae*	81	16.5(13.5 - 20.1)	1	16.7 (3.0 - 56.4)	3	9.7 (3.3 - 24.9)	0	0.0 (-)	7	4.4 (2.1 - 8.8)	14	1.4 (0.9 - 2.4)	106	6.0 (5.0 - 7.2)	7 (12.6)	8.3 (0.8 - 15.9)	<0.0005
Other/unspecified *Streptococcus* species	19	3.9 (2.5 - 6.0)	0	0.0 (-)	10	32.3 (18.6 - 49.9)	0	0.0 (-)	1	0.6 (0.1 - 3.5)	44	4.5 (3.4 - 6.0)	74	4.2 (3.4 - 5.2)	2.3 (4.2)	4.1 (0.9 - 7.3)	<0.0005
**Potentially pathogenic Gram-positives**	**269**	**54.9 (50.5 - 59.2)**	**1**	**16.7 (3.0 - 56.4)**	**15**	**48.4 (32.0 - 65.2)**	**38**	**36.9 (28.2 - 46.5)**	**36**	**22.5 (16.7 - 29.6)**	**243**	**25.0 (22.4 - 27.8)**	**602**	**34.2 (32.0 - 36.4)**	31 (22.4)	34.8 (20.7 - 49.0)	<0.0005
*Klebsiella* species	42	8.6 (6.4 - 11.4)	0	0.0 (-)	2	6.5 (1.8 - 20.7)	20	19.4 (12.9 - 28.1)	72	45.0 (37.5 - 52.7)	143	14.7 (12.6 - 17.1)	279	15.8 (14.2 - 17.6)	11.6 (11.3)	18.6 (9.9 - 27.3)	<0.0005
*Escherichia coli*	64	13.1 (10.4 - 16.3)	0	0.0 (-)	3	9.7 (3.3 - 24.9)	38	36.9 (28.2 - 46.5)	18	11.3 (7.2 - 17.1)	243	25.0 (22.4 - 27.8)	366	20.8 (19.0 - 22.7)	12.2 (12)	19.1 (11.1 - 27.1)	<0.0005
*Pseudomonas* species	22	4.5 (3.0 - 6.7)	0	0.0 (-)	4	12.9 (5.1 - 28.9)	4	3.9 (1.5 - 9.6)	18	11.3 (7.2 - 17.1)	140	14.4 (12.3 - 16.8)	188	10.7 (9.3 - 12.2)	7.9 (8.5)	9 (3.5 - 14.5)	<0.0005
*Enterobacter* species	5	1.0 (0.4 - 2.4)	0	0.0 (-)	4	12.9 (5.1 - 28.9)	1	1.0 (0.2 - 5.3)	3	1.9 (0.6 - 5.4)	56	5.8 (4.5 - 7.4)	69	3.9 (3.1 - 4.9)	1.4 (3.8)	2.9 (0.2 - 5.6)	<0.0005
*Serratia* species	0	0.0 (-)	0	0.0 (-)	0	0.0 (-)	1	1.0 (0.2 - 5.3)	0	0.0 (-)	39	4.0 (3.0 -5.4)	40	2.3 (1.7 - 3.1)	0 (0.7)	N/a (only 2 studies) -	-
*Proteus* species	9	1.8 (1.0 - 3.5)	0	0.0 (-)	0	0.0 (-)	1	1.0 (0.2 - 5.3)	0	0.0 (-)	3	0.3 (0.1 - 0.9)	13	0.7 (0.4 - 1.3)	0.2 (0.8)	0.5 (0.1 - 0.8)	0.07
*Salmonella* species	17	3.5 (2.2 - 5.5)	0	0.0 (-)	0	0.0 (-)	0	0.0 (-)	3	1.9 (0.6 - 5.4)	7	0.7 (0.3 - 1.5)	27	1.5 (1.1 - 2.2)	0.4 (1.6)	1.9 (0.0 - 3.9)	0.006
*Haemophilus influenzae*	25	5.1 (3.5 - 7.4)	3	50.0 (18.8 - 81.2)	1	3.2 (0.6 - 16.2)	0	0.0 (-)	1	0.6 (0.1 - 3.5)	2	0.2 (0.1 - 0.7)	32	1.8 (1.3 - 2.6)	1.9 (4.3)	2.3 (0.0 - 5.3	<0.0005
*Neisseria meningitidis*	11	2.2 (1.3 - 4.0)	2	33.3 (9.7 - 70.0)	0	0.0 (-)	0	0.0 (-)	0	0.0 (-)	0	0.0 (-)	13	0.7 (0.4 - 1.3)	0 (1.7)	N/a (only 2 studies)	-
*Acinetobacter* species	26	5.3 (3.7 - 7.7)	0	0.0 (-)	2	6.5 (1.8 - 20.7)	0	0.0 (-)	9	5.6 (3.0 - 10.3)	95	9.8 (8.1 - 11.8)	132	7.5 (6.4 - 8.8)	5.5 (4.9)	7 (4.0 - 10.0)	0
**Potentially pathogenic Gram-negatives**	**221**	**45.1(40.8 - 49.6)**	**5**	**83.3 (43.4 - 97.0)**	**16**	**51.6**	**65**	**63.1**	**124**	**77.5 (70.4 - 83.3)**	**728**	**75.0 (72.2 - 77.6)**	**1159**	**65.8 (63.6)**	69 (22.4)	65.2 (51.1 - 79.3)	<0.0005
**Total**	**490**	**100.0 (n/a)**	**6**	**100.0 (n/a)**	**31**	**100**	**103**	**100.0 (n/a)**	**160**	**100.0 (n/a)**	**971**	**100.0 (n/a)**	**1761**	**100.0 (n/a)**			

95% CI – 95% confidence interval, IQ range – interquartile range

**Figure 3:**
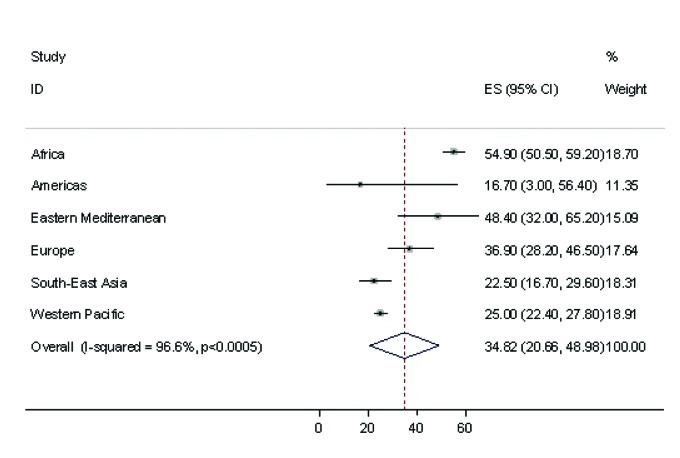
Forest plot of the summary estimate and 95% confidence interval of the prevalence of potentially pathogenic Gram-positives. Analysis is based on 27 studies in the 5 WHO regions (Africa: 11 studies, Americas: 1 study, Europe: 2 studies, Eastern Mediterranean: 1 study, South East Asia: 7 studies and Western Pacific: 5 studies). Weights are from random effects analysis. (ES: estimate, 95% CI: 95% confidence interval; I-squared and p-value are measures for heterogeneity between the studies).

**Figure 4:**
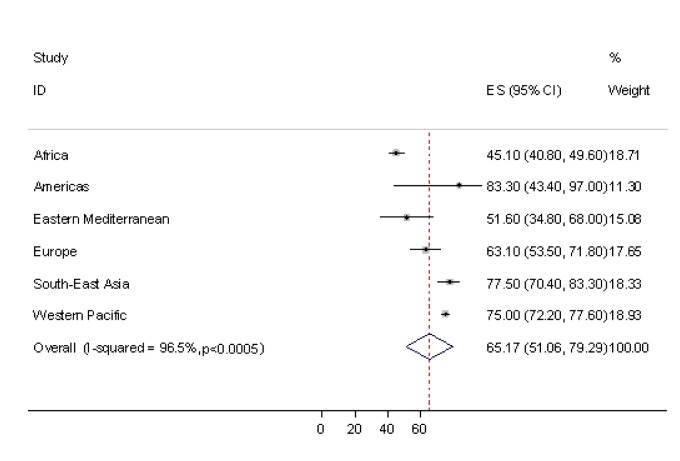
Forest plot of the summary estimate and 95% confidence interval of the prevalence of potentially pathogenic Gram-negatives. Analysis is based on 27 studies in the 5 WHO regions (Africa: 11 studies, Americas: 1 study, Europe: 2 studies, Eastern Mediterranean: 1 study, South East Asia: 7 studies and Western Pacific: 5 studies). Weights are from random effects analysis. (ES: Estimate, 95% CI: 95% confidence interval; I-squared and p-value are measures for heterogeneity between the studies).

**Figures 3**** and ****4** present the meta-analysis forest plot graphs for the potentially pathogenic Gram-positive and Gram-negative categories respectively. Forest plot graphs for all other potential pathogens are presented in **Supplementary Table 5**(Supplementary Table 5).

**Figure 5:**
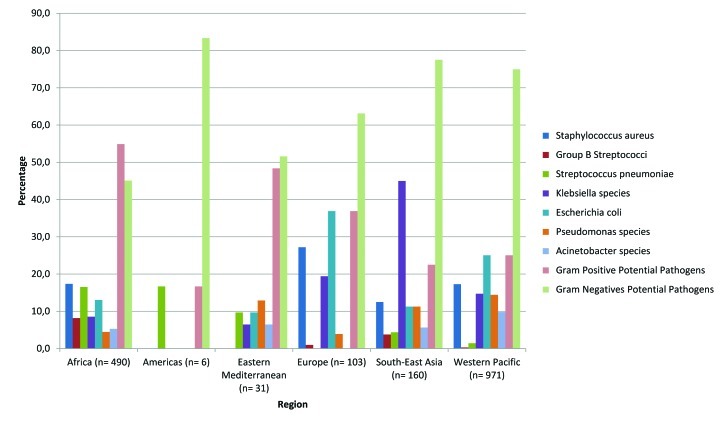
Percentage of selected potential pathogens in WHO regions. Numbers in parentheses indicate the total numbers of potential pathogens isolated for each region.

**Figure 6:**
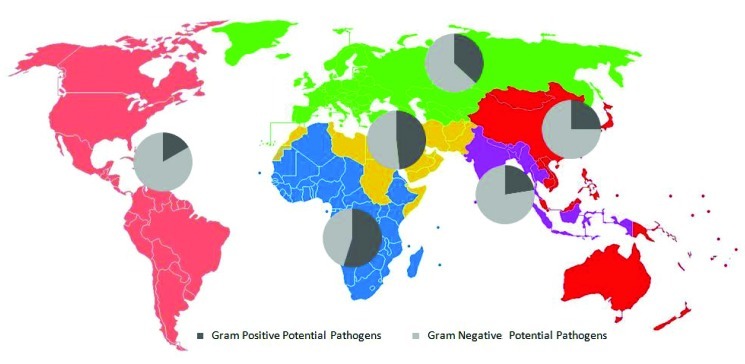
Distribution of Gram-positive and Gram-negative potential pathogens by region.

The percentages from **Table 4** on the six most commonly isolated organisms along with Group B *Streptococci* (GBS) are then illustrated in **Figure 5**. Group B *Streptococci* was included despite its relatively low prevalence so as to provide comparison with known high colonization rates experienced in many developed countries (57). **Figure 6** displays data from **Table 4** of the relative proportions of gram-positive and gram-negative potential pathogens in each region.

**Table 5** presents data of all organisms isolated in all regions categorised by different age-of-onset categories. Region-specific age-of-onset data for potential pathogens are presented in **Supplementary Table 3**(Supplementary Table 3). Eight studies from 6 countries in 4 regions (Africa, Europe, South-East Asia, Western Pacific) reported data for the category of neonates who were up to 7 days old. Data were presented in varying ways and in addition to aetiological data specified as sepsis occurring at ≤7 days of life, data reported as 4-6 days, 0-6 days, 0-7 days, 0-5 days and 'early-onset' were also included in this category.

**Table 5:** Distribution of all isolates by age-of-onset

	≤7 days of life		8-59 days of life		60-90 days of life	
Organism Isolated	N	% (95% CI)	N	% (95%CI)	N	% (95%CI)
*Staphylococcus aureus*	33	11.7 (8.0 - 15.5)	268	15.0 (13.4 - 16.7)	1	2.3 (0.0 - 6.7)
Group A *Streptococci/ Streptococcus pyogenes*	4	1.4 (0.0 - 2.8)	50	2.8 (2.0 -3.6)	8	18.2 (6.8 - 29.6)
Group B *Streptococci*	19	6.7 (3.8 - 9.7)	31	1.7 (1.1 - 2.4)	0	0.0 (-)
Group D *Streptococci/ Enterococcus*	4	1.4 (0.0 - 2.8)	13	0.7 (0.3 - 1.1)	0	0.0 (-)
*Streptococcus Pneumoniae*	13	4.6 (2.1 - 7.1)	93	5.2 (4.2 - 6.3)	14	31.8 (18.1 - 45.6)
Other/unspecified *Streptococcus* species	24	8.5 (5.3 - 11.8)	50	2.8 (2.0 - 3.6)	0	0.0 (-)
Other/ unspecified Gram-positives*	0	0.0 (-)	112	6.3 (5.2 - 7.4)	0	0.0 (-)
**All Gram-positives**	**97**	**34.4 (28.9 - 39.9)**	**617**	**34.6 (32.4 - 36.8)**	**23**	**52.3 (37.5 - 67.0)**
*Klebsiella pneumoniae*	22	7.8 (4.7 - 10.9)	232	13.0 (11.4 - 14.6)	2	4.5 (0.0 - 10.7)
Other/unspecified *Klebsiella* species	10	3.5 (1.4 - 5.7)	15	0.8 (0.4 - 1.3)	0	0.0 (-)
*Escherichia coli*	46	16.3 (12.0 - 20.6)	320	17.9 (16.2 - 19.7)	1	2.3 (0.0 - 6.7)
*Pseudomonas* species	22	7.8 (4.7 - 10.9)	166	9.3 (8.0 - 10.7)	1	2.3 (0.0 - 6.7)
*Enterobacter* species	10	3.5 (1.4 - 5.7)	59	3.3 (2.5 - 4.1)	0	0.0 (-)
*Serratia* species	0	0.0 (-)	40	2.2 (1.6 - 2.9)	1	2.3 (0.0 - 6.7)
*Proteus* species	6	2.1 (0.4 - 3.8)	7	0.4 (0.1 - 0.7)	0	0.0 (-)
*Salmonella* species	1	0.4 (0.0 - 1.0)	26	1.5 (0.9 - 2.0)	6	13.6 (3.5 - 23.8)
*Haemophilus influenzae*	2	0.7 (0.0 - 1.7)	30	1.7 (1.1 - 2.3)	4	9.1 (0.6 - 17.6)
*Neisseria meningitidis*	0	0.0 (-)	13	0.7 (0.3 -1.1)	0	0.0 (-)
*Acinetobacter* species	19	6.7 (3.8 - 8.7)	113	6.3 (5.2 - 7.5)	3	6.8 (0.0 - 14.3)
Other/unspecified Gram-negatives**	42	14.9 (10.7 - 19.0)	59	3.3 (2.5 - 4.1)	1	2.3 (0.0 - 6.7)
**All Gram-negatives**	**180**	**63.8 (58.2 - 69.4)**	**1080**	**60.5 (58.3 - 62.8)**	**19**	**43.2 (28.5 - 57.8)**
Non-stated/Undetermined	5	1.8 (0.2 - 3.3)	87	4.9 (3.9 - 5.9)	2	4.5 (0.0 - 10.7)
**Totals**	**282**	**100.0 (n/a)**	**1784**	**100.0 (n/a)**	**44**	**100.0 (n/a)**

*Includes data for *Aerococcus* sp., *Bacillus* sp. and others.

** Includes data for *Citrobacter* sp., *Moraxella* sp., *Shigella* sp., *Aeromonas* sp. and others; Data was also extracted for coagulase-negative *Staphylococci*: ≤7 days of life – 3 isolates, 7-59 dyas of life – 880 isolates, 60-90 days of life – 0 isolates.

However, all studies (from 14 countries, and in all regions) reported data for the category of neonates and young infants who were 8-59 days old. In addition to aetiological data specified as sepsis occurring at 8-59 days of life, data categorised as <2 months, 1-2 months, 8-60 days, 8-30 days, 7-59 days, 7-55 days, 7-28 days and ‘late-onset’ or being from ‘neonates/newborns’ were also included. Finally, five studies from five countries in two regions (Africa, Western Pacific) reported data for the category of infants 60-90 days of age. In addition to aetiological data specified as sepsis occurring at 60-90 days of life, data categorised as 2-3 months, 31-90 days, 30-90 days, 30-59 days and 1-3 months were also included.

### Study denominator

**Figure 7:**
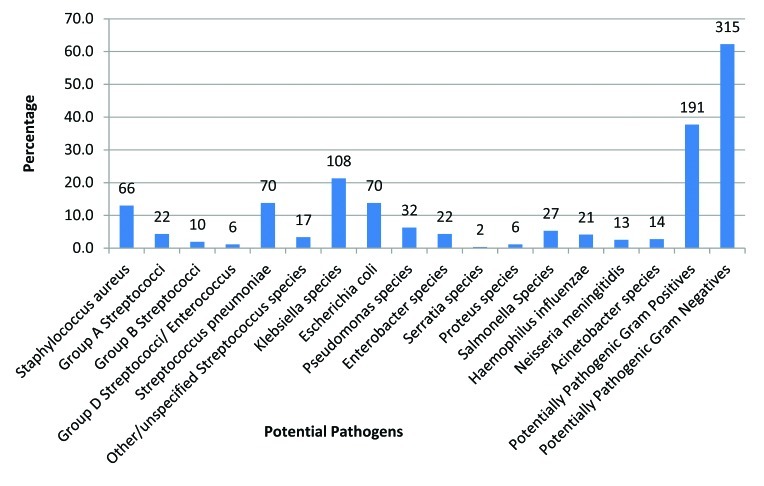
Potential pathogens in studies using patients as denominator. Data labels indicate the number of isolates.

**Figure 8:**
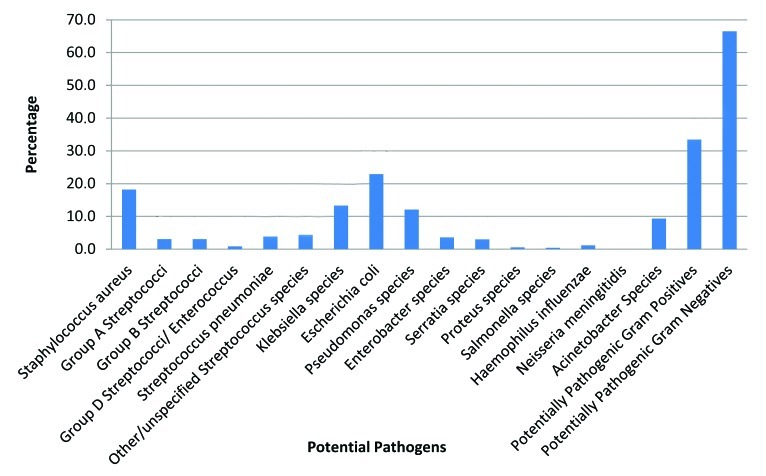
Potential pathogens in studies using isolates as denominator. Data labels indicate the number of isolates.

Nineteen studies used patients as a denominator, while 8 used isolates. Data on potential pathogens from all age-of-onset categories and regions were split into those 2 groupings and displayed in **Figures 7** and **8**, respectively.

### Other reported information

Ten studies from 8 countries in four regions reported data on CANS-specific case fatality rates, and these are detailed in **Table 6** which furthermore contains CANS-specific incidence data reported by 3 studies. With regard to the commonly reported risk factors for neonatal sepsis among premature or low birth weight neonates, only a single source of data was identified for each. Gatchalian and coworkers reported 46% incidence of CANS in low birth weight infants in low resource settings (43), while Mondal and coworkers quoted 22% of CANS incidence occurring in preterm neonates (38). Culture positivity rates varied significantly, with reported rates as high as 65% (41) and as low as 5% (26), or even 3% for some age groups (24). This could suggest significant differences among studies in inclusion criteria, case definition or capacity for accurate microbiological analysis, and therefore potentially data quality. Finally, 6 studies reported data concerning CANS-specific antimicrobial sensitivity patterns. Sensitivity percentages to selected antimicrobials for the most prevalent pathogens are displayed in **Table 7**.

**Table 6:** Reported case fatality rates and incidence data

Region	Article	Case fatality rate reported*	Incidence data reported
Africa	Berkley *et al* (22)	56% of ≤7 days of life, 26% of 8-59 days of life	1457/10^5^ person years (for infants <1year old)
	Mulholland *et al* (28)	31% of 0-91 days of life	—
	English *et al* (24)	27% of ≤7 days of life, 5% of 8-59 days of life	—
	Muhe *et al* (27)	49% of 0-59 days of life	—
	Campagne *et al* (23)	58% of 0-59 days of life	—
Americas	Weiss *et al* (30)	—	421/10^5^ person years (for infants <2months old)
South-East Asia	Mondal *et al* (38)	32% of 0-59 days of life	—
	Mathur *et al* (37)	70% of 0-59 days of life	—
Europe	Taskin *et al* (33)	5% of ≤7 days of life, 3% of 8-59 days of life	—
Western Pacific	Quiambao *et al* (45)	29% of 0-91 days of life	—
	Gatchalian *et al* (43)	26% of 0-91 days of life	—
	Choo *et al* (42)	—	1571/10^5^ live births

*Data were standardised to fit with age categories used in review.

**Table 7:** Percentage sensitivity patterns of most prevalent pathogens to selected antimicrobials

	Region	Africa		South-East Asia			
Organism	Antimicrobial	Adejuyigbe *et al* (20)	Muhe *et al* (27)	Mathur *et al* (37)	Panigrahi *et al* (39)	Darmstadt *et al* (35)	Tallur *et al* (41)
*Escherichia coli*	Amoxycillin (AMX)	60.0	—	—	—	—	—
	Ampicillin (AMP)	40.0	100.0	—	—	100.0	29.0
	Cefotaxime (CTX)	—	—	—	—	—	100.0
	Ceftazidime (CAZ)	—	100.0	—	—	100.0	—
	Ceftriaxone (CRO)	—	—	—	—	100.0	100.0
	Ciprofloxacin (CIP)	—	—	—	—	100.0	—
	Gentamicin (GEN)	80.0	100.0	—	—	100.0	71.0
	Imipenem (IMP)					100.0	—
*Staphylococcus aureus*	Amoxycillin (AMX)	73.0	—	—	—	—	—
	Ampicillin (AMP)	—	—	—	—	0.0	21.0
	Cefotaxime (CTX)	—	—	—	—	—	—
	Ceftazidime (CAZ)	—	—	—	—	66.7	—
	Ceftriaxone (CRO)	—	—	—	—	90.0	—
	Ciprofloxacin (CIP)	—	—	—	—	80.0	—
	Gentamicin (GEN)	85.8	—	—	—	90.0	29.0
	Imipenem (IMP)	—	—	—	—	90.0	—
*Klebsiella* species*	Amoxycillin (AMX)	0.0	—	—	—	—	—
	Ampicillin (AMP)	—	—	10.0	—	0.0	25.5
	Cefotaxime (CTX)	—	—	—	—	—	76.5
	Ceftazidime (CAZ)	—	—	—	22.0	33.3	—
	Ceftriaxone (CRO)	—	—	71.4	—	33.3	81.0
	Ciprofloxacin (CIP)	—	—	64.8	11.0	66.7	—
	Gentamicin (GEN)	100.0	—	42.8	—	66.7	59.5
	Imipenem (IMP)	—		100.0	—	100.0	—

*Averages were taken when more than one variant’s sensitivity patterns were reported.

## DISCUSSION

The overall data on CANS in low and middle income countries are very limited, with only 27 studies suitable for inclusion, many of which had small sample sizes and provided little data. This suggests a degree of caution over the overall validity of the review’s results. There was also significantly fewer data for the age-of-onset categories ≤7 days of life and 60-90 days of life. The geographic focus of studies in certain global regions is another concern, with 17 of 28 studies taking place in Africa or South-East Asia regions. Although the majority of studies being conducted in Africa can be argued to fit with Africa having the highest neonatal mortality rates globally, it should be noted that seven out of ten African studies were based in Nigeria or Kenya, and there were little or no data for other countries with comparable neonatal mortality levels. Similarly, 6 out of 7 South-East Asian studies were conducted in India, which again has high neonatal mortality rates. However, these are reportedly lower than in Pakistan, yet no studies were found from Pakistan (58).

The largest numbers of studies found by this review were conducted between 1991 and 1995 and only 2 studies were relevant to the most recent period of 2006-2010, highlighting the need for new research in this area and implying potential issues with the representativeness of data presented here. Excluding studies before 1980 to narrow the literature review may have resulted in missing some relevant studies. Although the search was systematic, some studies after 1980 may have also been missed due to the potential of human error in screening results. In addition, several foreign language studies were excluded because it was not possible to extract enough information to include them in the review. Several studies had very low culture positivity rates, resulting in small numbers of organisms being reported. The potential to generalise results is limited by their small sample sizes. Some studies presented considerably larger numbers of isolated organisms than others, thereby giving greater weight to their reported aetiological data.

To analyse aetiological data by the age-of-onset, it was necessary to standardise data into specific categories; however, these categories did not always fit with reported data. In certain cases it was therefore necessary to reassign and impute data. This was not the case for the significant majority of studies. Despite some of the limitations described above, data presented in this review should be generally robust and useful for planning international child health policy on tackling neonatal infections.

### Study design

Criteria for diagnosing neonatal sepsis varied significantly, providing inconsistencies in data and potential biases. Some studies included pneumonia and meningitis in this category, while others considered them indistinguishable from the data for sepsis. The criteria for excluding nosocomial infections also differed, with several studies merely separating data into babies born in the study hospital and babies born elsewhere (and other studies not defining criteria at all). The age-of-onset categories also were not fully consistent between studies, and there were some discrepancies with definitions of ‘early-onset’ and ‘late-onset’ sepsis. Several studies did not collect data for all age-of-onset categories, therefore not providing a complete picture of CANS aetiology. Also, a certain number of studies split data into community-acquired and nosocomial, and into different age-of-onset categories, but they did not combine these two categorisations. Still, in all cases we used the information from other studies to impute the data and align it with the majority of studies, in order to prevent valuable information from being lost.

Although several studies explicitly reported excluding infants who had received prior antimicrobial therapy, the majority of studies did not report inclusion or exclusion criteria relating to this factor. This is potentially an important issue as antimicrobial therapy prior to blood cultures being taken could significantly change aetiological patterns and therefore bias the reported data. Most studies reported data from babies who presented to primary facilities or outpatient services of referral facilities. The aetiological distribution may differ from that of babies who are born at home and die before reaching hospital. Only two studies adopted a community-surveillance approach that could counter this issue.

Culture positivity rates were reported to be considerably low by some studies, potentially indicating inadequate laboratory facilities or high prior antimicrobial use. Future reviews could potentially exclude these studies, but this would further decrease the amount of data. It is likely that the prevalence of certain pathogens such as *Haemophilus influenzae* is underestimated in many studies due to the significant diagnostic capacity required to isolate these organisms (4). For the same reason, it is possible that prevalence of less fastidious organisms such as *S. aureus* is overestimated, due to comparative ease of isolation. This is reinforced by a study that used the non-culture technique of antigen detection and reported significantly higher *H. influenzae* prevalence compared with many other studies (30).

### Distribution of CANS pathogens in developing countries

This review suggests that the majority of organisms that cause CANS in low and middle income countries are Gram-negative pathogens. The most commonly isolated are, in ranked order, *S. aureus, E coli* and *Klebsiella* species. These results are similar to a previous review, where the order of prevalence was *Klebsiella* species, *E coli* and *S. aureus* (4). The potential for significant comparisons of the regional distribution of potential pathogens is limited due to the paucity of data, particularly in the Eastern Mediterranean and Americas regions. Both Europe and Western Pacific regions are similar to the overall distribution with *S. aureus, E coli* and *Klebsiella* species the most prevalent organisms. The Western Pacific region also displays a higher prevalence of *Pseudomonas* species compared with overall aggregates. Europe has a high prevalence of Group D *Streptococci*. In the African region, *S. aureus* is seen as the most prevalent potential pathogen, followed by *Streptococcus pneumoniae*, which is only marginally less prevalent and is found in all age-of-onset categories. Notably, the prevalence of both Group A and Group B *Streptococci* is more than doubled in comparison to other regions. In the African region, unlike in any other region, 54.9% of potential pathogens isolated were Gram-positive. The Eastern Mediterranean region also showed a relatively high prevalence with 48% Gram-positive potential pathogens. This is important to consider, as some antibiotics are more effective against one than the other. South-East Asian region displayed a major predominance of *Klebsiella* species, followed by *S. aureus, E coli* and *Pseudomonas* species.

Although the distribution of potential pathogens is broadly similar in ≤7 days of life and 8-59 days of life, GBS isolates are notably more prevalent in the ≤7 days of life category. This was potentially expected, as neonatal GBS infection is commonly taken to be maternally-acquired (59). This review reports a lower prevalence of GBS as a CANS pathogen in developing countries than previous research (4). However, the prevalence of GBS neonatal sepsis in the first 7 days of life estimated by both this paper and previous review (4) is considerably lower than in developed countries (60). It is unclear whether a genuine aetiological difference is present, or there is a bias because most cases of early-onset sepsis in developing countries are not being registered because infants die before reaching health facilities (61). This is potentially supported by Stoll and Schuchat, who reported similar rates of maternal GBS colonization in developing countries compared with developed countries (62). Culture-negative GBS neonatal sepsis has also been shown in previous studies to be high, potentially as a result of maternal antibiotic therapy (63). This could result in underestimation of the burden of GBS neonatal sepsis if based on bacterial isolates.

The predominance of Gram-negative organisms and overall similar distribution of pathogens in the ≤7 days of life and the 8-59 days of life age-of-onset categories may contradict the assumption that early-onset neonatal sepsis is mainly maternally acquired. One potential explanation for these similar distributions is bias resulting from the necessary standardisation of primary data for analysis. However, this notion was also supported by another systematic review (4) and therefore may potentially have implications for CANS prevention, highlighting the need for improved hygienic practices before, during and after birth. In addition, similar aetiologies between the two groups may suggest that WHO ‘young infant’ guidelines (7-59 days) for management of sepsis could also be used for sepsis occurring before this period.

Data for the post-young infant category of 60-90 days of life showed marked differences compared with that of earlier age-of-onset categories, justifying the 7-59 day age group defined in WHO ‘young infant’ guidelines, because management is likely to be different for later ages-of-onset. There was a significantly higher relative prevalence of Group A *Streptococci*, *S. pneumoniae*, *Salmonella* species and *H. influenzae*, and an overall majority of Gram-positive organisms, as compared with the significant Gram-negative majorities in other categories. One potential reason for this may be a bias due to the small quantity of data for this category. However, similar aetiological distributions are reported by Zaidi et al. (4) and this notion could be of significance and relevant for informing antibiotic therapy.

### CANS compared with hospital-acquired neonatal sepsis

A recent review of hospital-acquired neonatal sepsis in developing countries showed a predominance of Gram-negative organisms with *Klebsiella* species most commonly isolated, followed by *S. aureus* and then *E coli* (5). This is similar to this review’s findings, suggesting potential similarities in major pathogens between CANS and hospital-acquired neonatal sepsis in developing countries. The hospital-acquired neonatal sepsis review, however, showed GBS as far more frequent causative organism than in this review of CANS. This may be due to easier capture of GBS in hospital-born babies as the vast majority of cases present within 48 hours of birth, whereas cases occurring in home-born babies may never have the opportunity to be diagnosed because of the fatal nature of this disease (60).

### Incidence, case-fatality, risk factors, diagnosis and treatment

CANS-specific incidence was reported in only 3 papers (3 countries, 3 regions), and in different formats, ranging from 421/100 000 person years for infants under 2 months to 1571/100 000 live births. These data are insufficient to draw more general conclusions, and a similar lack of data was highlighted in a recent review of the burden of neonatal infections in developing countries (64). Lack of health care access and low levels of care-seeking lead to significantly underestimation of CANS incidence in most studies (65), although community surveillance study designs may go some way to ameliorate this.

Data on Case Fatality Rates (CFRs) were also scarce, presented in only 10 studies (8 countries in 4 regions), with no data from the Americas and Eastern Mediterranean regions. The potential for comparison of CFRs is also limited as they are significantly dependant on age-of-onset and severity. However, the majority of studies reported CFRs of over 30%, with the overall CFR for developing countries seen to be as much as 20 times higher than that of developed countries. This is congruent with the estimate of 99% of neonatal deaths occurring in developing countries (1,60). Addressing reasons behind this gap and designing interventions to reduce its size are clear areas for policy development and implementation.

Comparing studies that used patients as a denominator with those that used isolates, the distribution of potential pathogens is generally similar. Studies using isolates displayed a higher prevalence of *S. aureus*, potentially reflecting multiple isolates for each patient rather than a difference in distribution. *S. pneumoniae* is significantly more prevalent in studies using patients as a denominator. This is most likely due to the fastidious nature of *S. pneumoniae* which quickly autolyses in blood cultures, whereas *S. aureus* is a hardy pathogen and is known to be associated with prolonged bacteremia in many cases (66,67).

Only 2 studies reported data concerning CANS-specific risk factors (38,43). Data reported suggests that low birth weight and prematurity are both significant risk factors for CANS, but further risk factors should also be evaluated.

Six studies from two regions reported CANS-specific data on pathogen antimicrobial sensitivity. Data reported suggests the main area of concern regarding antibiotic resistance is that of *Klebsiella* species, which did not have 100% of isolates sensitive to any antibiotic apart from imipenem, and had high rates of resistance to third generation cephalosporins and aminoglycosides. This is a significant point to consider for targeted antibiotic therapy, especially in the South-East Asian region where *Klebsiella* species account for 45% of CANS. There is a need for further surveillance to accurately determine the sensitivity patterns of CANS pathogens, thereby aiding appropriate therapy and minimising occurrence of resistance (17). Emerging antibiotic resistance is a global problem, and one potential reason for this is the wide availability of over-the-counter antibiotics in low and middle income countries (61). This is an important area to consider which to be combated requires both legislative and health promotion policies.

Coagulase-negative *Staphylococci* (CNS) were excluded as a contaminant in this and also in other reviews (4) and primary data sources (28), where no difference was reported in symptoms between patients with positive CNS blood cultures and those with entirely negative blood cultures (implying no pathogenicity). Conversely, in developed countries CNS are cited as major causative organisms of neonatal sepsis (60), although these data come from neonatal intensive care units and so further research is necessary to establish consensus on CNS pathogenicity with relation to CANS.

Overall data for the WHO Young Infants Study Group multicenter study (18) presented that 26% of *S. pneumoniae* isolates were type 2, which is not included in current pneumococcal conjugate vaccines. It is suggested that further research should be conducted in this area to establish this finding’s significance and inform future vacination development and policy (18).

### Implications for future research and international child health policy

This review has highlighted significant gaps in information concerning CANS in developing countries, and more research into this area is urgently required. Due to the small number of studies reporting CANS aetiology it is necessary to further evaluate results presented here, to determine whether they are specific to these studies or reflect genuine aetiological distributions. The number of studies presenting data concerning 2005-2010 is five times less than those presenting data for 2000-2010, which suggests that the interest in this type of research is decreasing.

Future research should focus on areas with high disease burden and a significant paucity of data, such as certain parts of South-East Asia and the Africa. Research into CANS occurring within the first 7 days of life is also significantly needed as approximately 75% of neonatal deaths occur within this period (58), yet aetiological data are particularly scarce. Based on characteristics and limitations of studies analysed in this review, it is recommended that all future studies into CANS aetiology in developing countries follow the minimum criteria presented in **Table 8**.

**Table 8:** Minimum preferred criteria for future research

Criteria	Preferred standard
Study design	Community surveillance
Case definition/ inclusion criteria	Use Darmstadt *et al* (35) algorithm for community-based neonatal assessment and diagnosis. Confirm microbiologically.
	Exclude infants if received prior antimicrobial therapy.
	Use explicit criteria for exclusion of nosocomial infection.
Data set	Use explicitly defined age-of-onset categories from WHO IMCI* guidelines.
	Report site-of-birth of all cases.
	Use highest feasible standard of microbiology facilities.
	Record incidence per 10^5^ live births in clearly defined study population.
	Record case fatality rates for microbiologically confirmed sepsis.
	Record data on risk factors.
	Study denominator should be patients.
	Test all isolates for antimicrobial sensitivity.

*WHO Integrated Management of Childhood Illness (16).

As a potential means of achieving greater and more representative data for CANS in developing countries the establishment of several community surveillance sites is recommended, equally situated in all WHO regions and providing coordinated multicentre research data using the criteria above (58). Significant investment would be required to ensure adequate surveillance, but this would be warranted because reducing neonatal mortality – particularly from infections - is crucial for progress towards Millennium Development Goal 4. Aetiological data from this review could be relevant when devising maternal and neonatal immunization strategies for developing countries. With mounting global levels of antibiotic resistance, immunization is becoming an increasingly important possible means of protecting neonates from infection (65). One potential approach is passive immunization through maternal vaccination for which vaccines against GBS, *S. pneumoniae* and *H. influenzae* are in development at present (68). Although vaccines against pathogens including Hepatitis B and Poliovirus are currently administered at birth in many countries, direct immunization of neonates is less well understood for the pathogens discussed in this review (69). There is also little current research into the use of established childhood vaccines such as pneumococcal or *H. influenzae* type-b (Hib) in the neonatal period, despite some positive previous indications (70). This review encourages more research in this area and also suggests the need for investigation into vaccination possibilities for other pathogens prevalent in CANS in developing countries. Data from this review specifically supports research into maternal and neonatal pneumococcal vaccination as *S. pneumoniae* was highly prevalent in Africa, including the very first week of life. Serological issues with the conjugate vaccine in neonates have however been highlighted above and these require further investigation.

This review also highlighted the need for identification, recognition and control of risk factors for CANS in developing countries. Low birth weight and prematurity are the only studied ones. Potential interventions to reduce the prevalence of these risk factors include improved maternal education and nutrition, prophylaxis/treatment of maternal malaria and other infections and overall improvement in socioeconomic conditions (71). Improved hygienic birth practices are also critical, emphasized by this review’s suggestion that similar aetiological distributions between age categories may actually mean that most of the infections are acquired from the environment. In community settings, improvements in hygienic practices can be linked to community and maternal health education and the training of traditional birth attendants (65).

High case fatality rates reported in this review highlight the critical need for early diagnosis of severe neonatal illness for improving outcomes. There is a clear need for the improvement of current diagnostic methods and development of novel methods which would be feasible within the low resources settings. The WHO Integrated Management of Childhood Illness (IMCI) guidelines (16) use diagnostic algorithms to assess illness, an approach that is highly indicated in community settings where diagnostic facilities are limited. A specific community-setting evaluation of a similar diagnostic algorithm showed high sensitivity and specificity (72), therefore suggesting that algorithms present a significant potential for providing accurate community-based diagnosis. Investments in dissemination of knowledge in this area and potential resultant policy approaches are encouraged. However, due to the varying aetiological distributions and antibiotic sensitivities found in this review, the development of effective low-cost pathogen detection techniques is also implicated as therapy informed by this is likely to be more effective. The case for improved diagnostics is further emphasized by poor levels of culture positivity and suggestions of potentially biased information due to difficulties in isolating fastidious organisms.

Knowledge on aetiology is essential for appropriate and effective treatment. The regional and age-of-onset aetiological variation presented in this review could be of use for local, national or regional bodies when devising case management guidelines. Further research would provide a more current picture of aetiological distribution and allow for regular guideline updates. The significant differences in aetiology between regions shown in this review suggest the benefits of a regional, rather than global, approach to case management guidelines. This review also reports potentially significant antimicrobial resistance levels, especially among *Klebsiella* species, further supporting the need for community surveillance sites as suggested above to monitor emerging resistance and inform attempts to minimize it. Due to the nature of CANS it is necessary to investigate the possibilities for effective low-cost treatment that is simple to administer in a community setting. For an antibiotic regimen to be appropriate to community settings, efficacy and safety even at an extended-interval dosing regimen is a desirable attribute (73). Penicillins and cephalosporins potentially fulfill those criteria. However, further evaluation of the efficacy of these and other antibiotics in community settings and against the spread of pathogens reported in this review is necessary to provide accurate recommendations of appropriate therapy for CANS.

There is noteworthy potential for the implementation of community-based care packages to reduce CANS incidence and resulting mortality. A trial in India involving hygienic birth practices, regular home visits, simple algorithms for detection of neonatal illness and referral to health care facilities, or community-based treatment using oral or parenteral antibiotic therapy, was shown to be highly effective (74). All interventions were conducted or overseen by trained community health personnel and were combined with community health education programmes including birth preparedness and promotion of preventative neonatal care practices. Based on comparison with control villages, the study reported a 70% reduction in neonatal mortality rates (74). This model has been replicated in other locations with similar successes, suggesting that its combination of community-based prevention, diagnosis and treatment could potentially provide a cost-effective and successful way of significantly reducing CANS incidence and mortality for many of the sites of studies analysed in this review (72,73,75-78). Implementation and evaluation of similar programmes could potentially be undertaken in conjunction with the community surveillance sites suggested above.

It must be noted that accurate data collection on aetiological distribution in low resource community settings can be a difficult task due to a lack of adequate microbiological facilities and trained staff (75). There are also considerable issues with the supply and quality of antimicrobials in certain developing areas (76) and a major requirement for accurate aetiological data and improved treatment in developing countries can be seen to be that of health system strengthening (77).

### Conclusion

This systematic literature review of community-acquired neonatal sepsis (CANS) in developing countries suggests that the most common causative pathogens are *S. aureus, E coli* and *Klebsiella* species, but with significant variation between regions and age-of-onset categories. This variation is important to monitor and consider for implementing appropriate therapy, devising management guidelines and informing related policy measures aimed at reducing CANS and overall neonatal mortality.

Several recommendations have been made to address issues highlighted by this paper. Data concerning the aetiology of CANS in developing countries are limited and significant future research is necessary, focusing on areas of high disease burden where there is a paucity of data. The establishment of community surveillance sites conducting coordinated research using minimum criteria is suggested to monitor CANS aetiology and chart antimicrobial sensitivity patterns. Other suggested areas of research include investigations into neonatal immunization, risk factors for CANS and development of effective low-cost diagnostics for improving microbiologic results. Health system strengthening is needed to enable positive improvements in accurate aetiological data and CANS prevention and management. A reduction in overall neonatal mortality rates is important for achieving Millennium Development Goal 4 and there is a significant potential for the implementation of community-based care practices to achieve this with relation to CANS.
