# Supplementary material for: Aetiology of community-acquired neonatal sepsis in low and middle income countries
Source: J Glob Health. 2011 Dec;1(2):154–70. (PMC3484773)
Supplement: Supplementary Table 1 [file jogh-01-154-s001.pdf]

## **Supplementary Table 1.** Full study characteristics

| Region | Author | Regional location | Specific location | Years of study | Duration of study | Culture/Sub Category | Study denominator | No. positive isolates | No. potentially pathogenic isolates |
|---|---|---|---|---|---|---|---|---|---|
| **Africa** | Adejuyigbe *et al* | Ile-Ife, Nigeria | Outpatient Health Centre | 2001 | 5 months | Blood/Swabs | Isolates | 54 | 37 |
| | Ayoola *et al* | Ibadan, Nigeria | Children's emergency ward, tertiary referral | 1998 | 5 months | Blood | Patients | 5 | 5 |
| | Berkley *et al* | Kilifi District, Kenya | Rural 1st referral level | 1998-2002 | 48 months | Blood | Isolates | 241 | 199 |
| | Campagne *et al* | Niamey, Nigeria | Central Laboratory Registers | 1981-1996 | 178 months | CSF | Patients | 101 | 58 |
| | English *et al* | Kilifi District, Kenya | Rural 1st referral level | 1999-2001 | 18 months | Blood/CSF | Isolates | 86 | 86 |
| | Ghiorgis *et al* | Addis Ababa, Ethiopia | Neonatal Unit | 1992-1993 | 21 months | Blood | Patients | 29 | 18 |
| | Herbert *et al* | Kilifi District, Kenya | Rural 1st referral level | 2004-2005 | 5 months | CSF | Isolates | 6 | 5 |
| | Muhe *et al* | Addis Ababa, Ethiopia | Primary and tertiary | 1991-1993 | 24 months | Blood/CSF | Patients | 41 | 41 |
| | Mulholland *et al* | Banjul, The Gambia | Primary and referral | 1990-1992 | 24 months | Blood/CSF | Patients | 55 | 54 |
| | Ojukwu *et al* | Abakaliki, Nigeria | NICU, Tertiary | 2002-2003 | 17 months | Blood | Patients | 14 | 14 |
| **Americas** | Weiss *et al* | Campinas, Brazil | Health Department Surveillance | 1997-1998 | 12 months | Blood/CSF/ Antigen detection | Patients | 33 | 6 |
| **Eastern Mediterran-ean** | Aletayeb *et al* | Khuzestan, Iran | Neonatal Unit, Referral | 1997-2007 | 120 months | CSF | Patients | 14 | 14 |
| | Maalej *et al* | Sfax, Tunisia | University Hospital | 1993-2001 | 96 months | CSF | Patients | 28 | 17 |
| **Europe** | Taskin *et al* | Turkey | NICU | Not reported | Not Reported | Blood/CSF/Urine | Isolates | 71 | 62 |
| | Biyikli *et al* | Istanbul, Turkey | University Hospital | 1999-2002 | 36 months | Urine | Patients | 44 | 44 |

| | | | | | | | | | |
|---|---|---|---|---|---|---|---|---|---|
| **South-East Asia** | Darmstadt *et al* | Mirzapur, Bangladesh | Community Surveillance + Rural Primary | 2004-2006 | 36 months | Blood | Isolates | 30 | 29 |
| | Das *et al* | Pondicherry, India | Referral | 1994-1995 | 18 months | Blood/CSF | Patients | 43 | 7 |
| | Mathur *et al* | New Delhi, India | Neonatal Unit, Referral | 2005 | 11 months | Blood | Patients | 20 | 20 |
| | Mondal *et al* | Pondicherry, India | Referral | 1988-1989 | 12 months | Blood | Patients | 16 | 13 |
| | Panigrahi *et al* | Orissa, India | Community Surveillance + Referral | Not Reported | not reported | Blood/CSF | Patients | 59 | 27 |
| | Sahai *et al* | Pondicherry, India | Tertiary, Urban | 1994- 1996 | 19 months | CSF | Patients | 5 | 5 |
| | Tallur *et al* | Karnataka, India | NICU, Rural | 1996-1997 | 16 months | Blood | Patients | 54 | 42 |
| **Western Pacific** | Choo *et al* | Kelantan, Malaysia | Neonatal Unit, Rural tertiary referral | 1985-1986 | 12 months | Blood | Patients | 54 | 42 |
| | Gatchalian *et al* | Manila, Philippines | Government, Community and Referral | 1991-1993 | 24 months | Blood/CSF | Patients | 35 | 35 |
| | Lehmann *et al* | Papua New Guinea | Outpatient department | 1991-1993 | 24 months | Blood | Isolates | 48 | 48 |
| | Quiambao *et al* | Bohol, Philippines | Rural 1st referral level | 1994-2000 | 72 months | Blood/CSF | Patients | 34 | 34 |
| | Yu *et al* | Sichuan, China | Neonatal Ward, referral | 1987-1998 | 24 months | Blood and local infections | Isolates | 1765 | 840 |
