# Supplementary material for: Aetiology of community-acquired neonatal sepsis in low and middle income countries
Source: J Glob Health. 2011 Dec;1(2):154–70. (PMC3484773)
Supplement: Supplementary Table 2 [file jogh-01-154-s002.pdf]

Supplementary Table 2. Individual study tables

**Adejuyigbe *et al* (2004)**	Age category (actual age range of the study)		
**Organism isolated**	≤7 days of life	8-59 days of life	60-90 days of life
		7-55 days	
Staphylococcus aureus		28	
Coagulase negative Staphylococci		17	
Group A Streptococci/ *Streptococcus Pyogenes*			
Group B Streptococci			
Group D Streptococci/ *Enterococcus*			
Group G Streptococci			
Streptococcus pneumoniae			
Other/unspecified *Streptococcus* species			
Other/ unspecified Gram positives			
**All Gram positives**	0	45	0
Klebsiella pneumoniae		1	
Other/unspecified *Klebsiella* species			
Escherichia coli		1	
Pseudomonas species		2	
Enterobacter species		1	
Serratia species			
Proteus species		3	
Salmonella species		1	
Citrobacter species		0	
Haemophilus influenzae			
Neisseria meningitidis			
Acinetobacter species			
Moraxella species			
Other/unspecified Gram negatives			
**All Gram negatives**	0	9	0
Non-stated/Undetermined			
**Totals**	0	54	0
**TOTAL**			54

**Aletayeb *et al* (2010)**			
**Organism Isolated**	≤7 days of life	8-59 days of life	60-90 days of life
		0-28 days	
Staphylococcus aureus		0	
Coagulase Negative Staphylococci			
Group A Streptococci/ *Streptococcus Pyogenes*			
Group B Streptococci			
Group D Streptococci/ *Enterococcus*		2	
Group G Streptococci			
Streptococcus pneumoniae			
Other/unspecified *Streptococcus* species			
Other/ unspecified Gram positives			
**All Gram positives**	0	2	0
Klebsiella pneumoniae		2	
Other/unspecified *Klebsiella* species			
Escherichia coli		3	
Pseudomonas species		1	
Enterobacter species		4	
Serratia species			
Proteus species			
Salmonella species			
Citrobacter species			
Haemophilus influenzae		0	
Neisseria meningitidis			
Acinetobacter species		2	
Moraxella species			
Other/unspecified Gram negatives			
**All Gram negatives**	0	12	0
Non-stated/Undetermined			
**Totals**	0	14	0
**TOTAL**			14

**Ayoola *et al* (2002)**			
**Organism Isolated**	≤7 days of life	8-59 days of life	60-90 days of life
		1-2 Months	
Staphylococcus aureus		1	
Coagulase Negative Staphylococci			
Group A Streptococci/ *Streptococcus Pyogenes*			
Group B Streptococci			
Group D Streptococci/ *Enterococcus*			
Group G Streptococci			
Streptococcus pneumoniae			
Other/unspecified *Streptococcus* species		0	
Other/ unspecified Gram positives			
**All Gram positives**	0	1	0
Klebsiella pneumoniae			
Other/unspecified *Klebsiella* species		1	
Escherichia coli		3	
Pseudomonas species		0	
Enterobacter species			
Serratia species			
Proteus species		0	
Salmonella species		0	
Citrobacter species			
Haemophilus influenzae			
Neisseria meningitidis			
Acinetobacter species			
Moraxella species			
Other/unspecified Gram negatives			
**All Gram negatives**	0	4	0
Non-stated/Undetermined			
**Totals**	0	5	0
**TOTAL**			5

**Berkley *et al* (2005)**			
**Organism Isolated**	≤7 days of life	8-59 days of life	60-90 days of life
Staphylococcus aureus	7	13	
Coagulase Negative Staphylococci			
Group A Streptococci/ *Streptococcus Pyogenes*	3	17	
Group B Streptococci	11	15	
Group D Streptococci/ *Enterococcus*			
Group G Streptococci			
Streptococcus pneumoniae	5	14	
Other/unspecified *Streptococcus* species	11	3	
Other/ unspecified Gram positives			
**All Gram positives**	37	62	0
Klebsiella pneumoniae	13	7	
Other/unspecified *Klebsiella* species			
Escherichia coli	25	8	
Pseudomonas species	6	3	
Enterobacter species			
Serratia species			
Proteus species			
Salmonella species	1	4	
Citrobacter species			
Haemophilus influenzae	1	7	
Neisseria meningitidis			
Acinetobacter species	16	9	
Moraxella species			
Other/unspecified Gram negatives	30	12	
**All Gram negatives**	92	50	0
Non-stated/Undetermined			
**Totals**	129	112	0
**TOTAL**			241

**Biyikli *et al* (2004)**			
**Organism Isolated**	≤7 days of life	8-59 days of life	60-90 days of life
		* 0-30 days	
Staphylococcus aureus		2	
Coagulase Negative Staphylococci			
Group A Streptococci/ *Streptococcus Pyogenes*			
Group B Streptococci		1	
Group D Streptococci/ *Enterococcus*		4	
Group G Streptococci			
Streptococcus pneumoniae			
Other/unspecified *Streptococcus* species			
Other/ unspecified Gram positives			
**All Gram positives**	0	7	0
Klebsiella pneumoniae		14	
Other/unspecified *Klebsiella* species			
Escherichia coli		20	
Pseudomonas species			
Enterobacter species		1	
Serratia species		1	
Proteus species		1	
Salmonella species			
Citrobacter species			
Haemophilus influenzae			
Neisseria meningitidis			
Acinetobacter species			
Moraxella species			
Other/unspecified Gram negatives			
**All Gram negatives**	0	37	0
Non-stated/Undetermined			
**Totals**	0	44	0
**TOTAL**			44

**Campagne *et al* (1999)**			
**Organism Isolated**	≤7 days of life	8-59 days of life	60-90 days of life
Staphylococcus aureus			
Coagulase Negative Staphylococci			
Group A Streptococci/ *Streptococcus Pyogenes*			
Group B Streptococci			
Group D Streptococci/ *Enterococcus*			
Group G Streptococci			
Streptococcus pneumoniae		34	
Other/unspecified *Streptococcus* species		3	
Other/ unspecified Gram positives			
**All Gram positives**	0	37	0
Klebsiella pneumoniae			
Other/unspecified *Klebsiella* species			
Escherichia coli			
Pseudomonas species			
Enterobacter species			
Serratia species			
Proteus species			
Salmonella species			
Citrobacter species			
Haemophilus influenzae		10	
Neisseria meningitidis		11	
Acinetobacter species			
Moraxella species			
Other/unspecified Gram negatives		15	
**All Gram negatives**	0	36	0
Non-stated/Undetermined		28	
**Totals**	0	101	0
**TOTAL**			101

**Choo *et al* (1988)**			
**Organism Isolated**	≤7 days of life	8-59 days of life	60-90 days of life
	0-5 days	*5-28 days*	
Staphylococcus aureus	6	11	
Coagulase Negative Staphylococci	3	11	
Group A Streptococci/ *Streptococcus Pyogenes*			
Group B Streptococci	1	0	
Group D Streptococci/ *Enterococcus*			
Group G Streptococci			
Streptococcus pneumoniae			
Other/unspecified *Streptococcus* species	2	1	
Other/ unspecified Gram positives			
**All Gram positives**	12	23	0
Klebsiella pneumoniae	5	2	
Other/unspecified *Klebsiella* species			
Escherichia coli	2	1	
Pseudomonas species	2	3	
Enterobacter species			
Serratia species			
Proteus species	1	1	
Salmonella species			
Citrobacter species			
Haemophilus influenzae			
Neisseria meningitidis			
Acinetobacter species			
Moraxella species			
Other/unspecified Gram negatives		1	
**All Gram negatives**	10	8	0
Non-stated/Undetermined	1		
**Totals**	23	31	0
**TOTAL**			54

**Darmstadt *et al* (2009)**			
**Organism Isolated**	≤7 days of life	8-59 days of life	60-90 days of life
	0-6 days	*7-27 days*	
Staphylococcus aureus	4	6	
Coagulase Negative Staphylococci			
Group A Streptococci/ *Streptococcus Pyogenes*			
Group B Streptococci	1	0	
Group D Streptococci/ *Enterococcus*			
Group G Streptococci			
Streptococcus pneumoniae	2	1	
Other/unspecified *Streptococcus* species	1	0	
Other/ unspecified Gram positives			
**All Gram positives**	8	7	0
Klebsiella pneumoniae	0	2	
Other/unspecified *Klebsiella* species	0	1	
Escherichia coli	1	0	
Pseudomonas species	2	3	
Enterobacter species	1	1	
Serratia species			
Proteus species			
Salmonella species			
Citrobacter species			
Haemophilus influenzae			
Neisseria meningitidis			
Acinetobacter species	2	1	
Moraxella species			
Other/unspecified Gram negatives	1	0	
**All Gram negatives**	7	8	0
Non-stated/Undetermined			
**Totals**	15	15	0
**TOTAL**			30

**Das *et al* (1998)**			
**Organism Isolated**	≤7 days of life	8-59 days of life	60-90 days of life
		Neonates	
Staphylococcus aureus		4	
Coagulase Negative Staphylococci		17	
Group A Streptococci/ *Streptococcus Pyogenes*			
Group B Streptococci		5	
Group D Streptococci/ *Enterococcus*			
Group G Streptococci			
Streptococcus pneumoniae		1	
Other/unspecified *Streptococcus* species			
Other/ unspecified Gram positives		1	
**All Gram positives**	0	28	0
Klebsiella pneumoniae		4	
Other/unspecified *Klebsiella* species			
Escherichia coli		3	
Pseudomonas species		3	
Enterobacter species		1	
Serratia species			
Proteus species			
Salmonella species		2	
Citrobacter species			
Haemophilus influenzae		0	
Neisseria meningitidis			
Acinetobacter species		2	
Moraxella species			
Other/unspecified Gram negatives			
**All Gram negatives**	0	15	0
Non-stated/Undetermined			
**Totals**	0	43	0
**TOTAL**			43

English *et al* (2003)			
**Organism Isolated**	≤7 days of life	8-59 days of life	60-90 days of life
		8-60 days	*61-90 days*
Staphylococcus aureus	1	4	1
Coagulase Negative Staphylococci			
Group A Streptococci/ *Streptococcus Pyogenes*	0	6	0
Group B Streptococci	6	6	0
Group D Streptococci/ *Enterococcus*	3	1	0
Group G Streptococci			
Streptococcus pneumoniae	3	7	3
Other/unspecified *Streptococcus* species			
Other/ unspecified Gram positives			
**All Gram positives**	13	24	4
Klebsiella pneumoniae			
Other/unspecified *Klebsiella* species	10	1	0
Escherichia coli	8	3	0
Pseudomonas species	5	1	1
Enterobacter species			
Serratia species			
Proteus species	4	0	0
Salmonella species			
Citrobacter species			
Haemophilus influenzae	0	4	1
Neisseria meningitidis			
Acinetobacter species			
Moraxella species			
Other/unspecified Gram negatives			
**All Gram negatives**	27	9	2
Non-stated/Undetermined	2	3	2
**Totals**	42	36	8
**TOTAL**			86

**Gatchalian *et al* (1999)**			
**Organism Isolated**	≤7 days of life	8-59 days of life	60-90 days of life
Staphylococcus aureus	1	2	0
Coagulase Negative Staphylococci			
Group A Streptococci/ *Streptococcus Pyogenes*	1	0	2
Group B Streptococci			
Group D Streptococci/ *Enterococcus*			
Group G Streptococci			
Streptococcus pneumoniae	0	2	1
Other/unspecified *Streptococcus* species			
Other/ unspecified Gram positives			
**All Gram positives**	2	4	3
Klebsiella pneumoniae	0	1	0
Other/unspecified *Klebsiella* species			
Escherichia coli	2	2	0
Pseudomonas species	2		
Enterobacter species	2	2	0
Serratia species			
Proteus species			
Salmonella species	0	4	2
Citrobacter species			
Haemophilus influenzae			1
Neisseria meningitidis			
Acinetobacter species	1	2	3
Moraxella species			
Other/unspecified Gram negatives	0	1	1
**All Gram negatives**	7	12	7
Non-stated/Undetermined			
**Totals**	9	16	10
**TOTAL**			35

**Ghiorgis (1997)**			
**Organism Isolated**	≤7 days of life	8-59 days of life	60-90 days of life
		Neonates/Newborns	
Staphylococcus aureus		1	
Coagulase Negative Staphylococci		11	
Group A Streptococci/ *Streptococcus Pyogenes*			
Group B Streptococci			
Group D Streptococci/ *Enterococcus*			
Group G Streptococci			
Streptococcus pneumoniae			
Other/unspecified *Streptococcus* species			
Other/ unspecified Gram positives			
**All Gram positives**	0	12	0
Klebsiella pneumoniae			
Other/unspecified *Klebsiella* species		7	
Escherichia coli		1	
Pseudomonas species		3	
Enterobacter species		1	
Serratia species			
Proteus species		1	
Salmonella species		3	
Citrobacter species		1	
Haemophilus influenzae			
Neisseria meningitidis			
Acinetobacter species		0	
Moraxella species			
Other/unspecified Gram negatives			
**All Gram negatives**	0	17	0
Non-stated/Undetermined			
**Totals**	0	29	0
**TOTAL**			29

**Herbert *et al* (2006)**			
**Organism Isolated**	≤7 days of life	8-59 days of life	60-90 days of life
		<2 Months	
Staphylococcus aureus			
Coagulase Negative Staphylococci			
Group A Streptococci/ *Streptococcus Pyogenes*		1	
Group B Streptococci			
Group D Streptococci/ *Enterococcus*			
Group G Streptococci			
Streptococcus pneumoniae		2	
Other/unspecified *Streptococcus* species		1	
Other/ unspecified Gram positives		1	
**All Gram positives**	0	5	0
Klebsiella pneumoniae			
Other/unspecified *Klebsiella* species			
Escherichia coli		0	
Pseudomonas species			
Enterobacter species			
Serratia species			
Proteus species			
Salmonella species		0	
Citrobacter species			
Haemophilus influenzae		0	
Neisseria meningitidis			
Acinetobacter species		1	
Moraxella species			
Other/unspecified Gram negatives			
**All Gram negatives**	0	1	0
Non-stated/Undetermined			
**Totals**	0	6	0
**TOTAL**			6

**Lehmann *et al* (1999)**			
**Organism Isolated**	≤7 days of life	8-59 days of life	60-90 days of life
		0-60 days	*61-90 days*
Staphylococcus aureus		10	0
Coagulase Negative Staphylococci			
Group A Streptococci/ *Streptococcus Pyogenes*		8	5
Group B Streptococci		1	
Group D Streptococci/ *Enterococcus*		2	0
Group G Streptococci		1	
Streptococcus pneumoniae		7	6
Other/unspecified *Streptococcus* species			
Other/ unspecified Gram positives			
**All Gram positives**	0	29	11
Klebsiella pneumoniae		1	1
Other/unspecified *Klebsiella* species			
Escherichia coli		3	0
Pseudomonas species			
Enterobacter species		1	0
Serratia species			
Proteus species			
Salmonella species			
Citrobacter species			
Haemophilus influenzae		1	1
Neisseria meningitidis			
Acinetobacter species			
Moraxella species			
Other/unspecified Gram negatives			
**All Gram negatives**	0	6	2
Non-stated/Undetermined			
**Totals**	0	35	13
**TOTAL**			48

**Maalej *et al* (2006)**			
**Organism Isolated**	≤7 days of life	8-59 days of life	60-90 days of life
		<1month	
Staphylococcus aureus		0	
Coagulase Negative Staphylococci			
Group A Streptococci/ *Streptococcus Pyogenes*			
Group B Streptococci			
Group D Streptococci/ *Enterococcus*			
Group G Streptococci			
Streptococcus pneumoniae		3	
Other/unspecified *Streptococcus* species		10	
Other/ unspecified Gram positives		0	
**All Gram positives**	0	13	0
Klebsiella pneumoniae			
Other/unspecified *Klebsiella* species			
Escherichia coli			
Pseudomonas species		3	
Enterobacter species			
Serratia species			
Proteus species			
Salmonella species			
Citrobacter species			
Haemophilus influenzae		1	
Neisseria meningitidis		0	
Acinetobacter species			
Moraxella species			
Other/unspecified Gram negatives		11	
**All Gram negatives**	0	15	0
Non-stated/Undetermined			
**Totals**	0	28	0
**TOTAL**			28

**Mathur *et al* (2010)**			
**Organism Isolated**	≤7 days of life	8-59 days of life	60-90 days of life
		Neonates/Newborns	
Staphylococcus aureus		2	
Coagulase Negative Staphylococci			
Group A Streptococci/ *Streptococcus Pyogenes*			
Group B Streptococci			
Group D Streptococci/ *Enterococcus*			
Group G Streptococci			
Streptococcus pneumoniae			
Other/unspecified *Streptococcus* species			
Other/ unspecified Gram positives			
**All Gram positives**	0	2	0
Klebsiella pneumoniae		14	
Other/unspecified *Klebsiella* species			
Escherichia coli		1	
Pseudomonas species		2	
Enterobacter species			
Serratia species			
Proteus species			
Salmonella species			
Citrobacter species			
Haemophilus influenzae			
Neisseria meningitidis			
Acinetobacter species		1	
Moraxella species			
Other/unspecified Gram negatives			
**All Gram negatives**	0	18	0
Non-stated/Undetermined			
**Totals**	0	20	0
**TOTAL**			20

**Mondal *et al* (1991)**			
**Organism Isolated**	≤7 days of life	8-59 days of life	60-90 days of life
		Neonates/Newborns	
Staphylococcus aureus		1	
Coagulase Negative Staphylococci		3	
Group A Streptococci/ *Streptococcus Pyogenes*		2	
Group B Streptococci			
Group D Streptococci/ *Enterococcus*			
Group G Streptococci			
Streptococcus pneumoniae			
Other/unspecified *Streptococcus* species			
Other/ unspecified Gram positives			
**All Gram positives**	0	6	0
Klebsiella pneumoniae		1	
Other/unspecified *Klebsiella* species			
Escherichia coli			
Pseudomonas species		2	
Enterobacter species			
Serratia species			
Proteus species			
Salmonella species			
Citrobacter species			
Haemophilus influenzae			
Neisseria meningitidis			
Acinetobacter species		3	
Moraxella species			
Other/unspecified Gram negatives			
**All Gram negatives**	0	6	0
Non-stated/Undetermined		4	
**Totals**	0	16	0
**TOTAL**			16

**Muhe *et al* (1999)**			
**Organism Isolated**	≤7 days of life	8-59 days of life	60-90 days of life
		0-2 Months	*2-3 Months*
Staphylococcus aureus		2	0
Coagulase Negative Staphylococci			
Group A Streptococci/ *Streptococcus Pyogenes*		9	0
Group B Streptococci			
Group D Streptococci/ *Enterococcus*			
Group G Streptococci			
Streptococcus pneumoniae		7	3
Other/unspecified *Streptococcus* species			
Other/ unspecified Gram positives			
**All Gram positives**	0	18	3
Klebsiella pneumoniae		1	0
Other/unspecified *Klebsiella* species			
Escherichia coli		10	0
Pseudomonas species			
Enterobacter species			
Serratia species		0	1
Proteus species			
Salmonella species		3	2
Citrobacter species			
Haemophilus influenzae		3	0
Neisseria meningitidis			
Acinetobacter species			
Moraxella species			
Other/unspecified Gram negatives			
**All Gram negatives**	0	17	3
Non-stated/Undetermined			
**Totals**	0	35	6
**TOTAL**			41

**Mulholland *et al* (1999)**			
**Organism Isolated**	≤7 days of life	8-59 days of life	60-90 days of life
Staphylococcus aureus	1	18	
Coagulase Negative Staphylococci			
Group A Streptococci/ *Streptococcus Pyogenes*		4	1
Group B Streptococci		2	
Group D Streptococci/ *Enterococcus*			
Group G Streptococci		1	
Streptococcus pneumoniae		9	1
Other/unspecified *Streptococcus* species			
Other/ unspecified Gram positives			
**All Gram positives**	1	34	2
Klebsiella pneumoniae			1
Other/unspecified *Klebsiella* species			
Escherichia coli	1	2	1
Pseudomonas species		1	
Enterobacter species	2	1	
Serratia species*			
Proteus species	1		
Salmonella species		4	2
Citrobacter species			
Haemophilus influenzae			1
Neisseria meningitidis			
Acinetobacter species			
Moraxella species			
Other/unspecified Gram negatives		1	
**All Gram negatives**	4	9	5
Non-stated/Undetermined			
**Totals**	5	43	7
**TOTAL**			55

* Age data for *Serratia* not reported, data redistributed based on global ratios

**Ojukwu *et al* (2005)**			
**Organism Isolated**	≤7 days of life	8-59 days of life	60-90 days of life
		0-28 days	
Staphylococcus aureus		9	
Coagulase Negative Staphylococci			
Group A Streptococci/ *Streptococcus Pyogenes*			
Group B Streptococci			
Group D Streptococci/ *Enterococcus*			
Group G Streptococci			
Streptococcus pneumoniae			
Other/unspecified *Streptococcus* species		0	
Other/ unspecified Gram positives			
**All Gram positives**	0	9	0
Klebsiella pneumoniae			
Other/unspecified *Klebsiella* species		1	
Escherichia coli		2	
Pseudomonas species		1	
Enterobacter species		0	
Serratia species			
Proteus species		0	
Salmonella species		1	
Citrobacter species			
Haemophilus influenzae		0	
Neisseria meningitidis			
Acinetobacter species			
Moraxella species			
Other/unspecified Gram negatives			
**All Gram negatives**	0	5	0
Non-stated/Undetermined			
**Totals**	0	14	0
**TOTAL**			14

Panigrahi *et al* (2004)			
**Organism Isolated**	≤7 days of life	8-59 days of life	60-90 days of life
		Neonates/Newborns	
Staphylococcus aureus			
Coagulase Negative Staphylococci		10	
Group A Streptococci/ *Streptococcus Pyogenes*			
Group B Streptococci			
Group D Streptococci/ *Enterococcus*			
Group G Streptococci			
Streptococcus pneumoniae			
Other/unspecified *Streptococcus* species			
Other/ unspecified Gram positives			
**All Gram positives**	0	10	0
Klebsiella pneumoniae		22	
Other/unspecified *Klebsiella* species			
Escherichia coli		5	
Pseudomonas species			
Enterobacter species			
Serratia species			
Proteus species			
Salmonella species			
Citrobacter species			
Haemophilus influenzae			
Neisseria meningitidis			
Acinetobacter species			
Moraxella species			
Other/unspecified Gram negatives			
**All Gram negatives**	0	27	0
Non-stated/Undetermined		22	
**Totals**	0	59	0
**TOTAL**			59

**Quiambao *et al* (2007)**			
**Organism Isolated**	≤7 days of life	8-59 days of life	60-90 days of life
	0-6	*7-59 days*	
Staphylococcus aureus	2	0	
Coagulase Negative Staphylococci			
Group A Streptococci/ *Streptococcus Pyogenes*	0	3	
Group B Streptococci	0	1	
Group D Streptococci/ *Enterococcus*			
Group G Streptococci			
Streptococcus pneumoniae	0	5	
Other/unspecified *Streptococcus* species			
Other/ unspecified Gram positives			
**All Gram positives**	2	9	0
Klebsiella pneumoniae	3	1	
Other/unspecified *Klebsiella* species			
Escherichia coli		3	
Pseudomonas species	2	1	
Enterobacter species	5	3	
Serratia species			
Proteus species	0	1	
Salmonella species	0	3	
Citrobacter species			
Haemophilus influenzae	0	1	
Neisseria meningitidis			
Acinetobacter species			
Moraxella species			
Other/unspecified Gram negatives			
**All Gram negatives**	10	13	0
Non-stated/Undetermined			
**Totals**	12	22	0
**TOTAL**			34

**Sahai *et al* (2001)**			
**Organism Isolated**	≤7 days of life	8-59 days of life	60-90 days of life
		1-3 Months	
Staphylococcus aureus			
Coagulase Negative Staphylococci			
Group A Streptococci/ *Streptococcus Pyogenes*			
Group B Streptococci			
Group D Streptococci/ *Enterococcus*			
Group G Streptococci			
Streptococcus pneumoniae		3	
Other/unspecified *Streptococcus* species			
Other/ unspecified Gram positives			
**All Gram positives**	0	3	0
Klebsiella pneumoniae			
Other/unspecified *Klebsiella* species			
Escherichia coli			
Pseudomonas species			
Enterobacter species			
Serratia species			
Proteus species			
Salmonella species		1	
Citrobacter species			
Haemophilus influenzae		1	
Neisseria meningitidis			
Acinetobacter species			
Moraxella species			
Other/unspecified Gram negatives			
**All Gram negatives**	0	2	0
Non-stated/Undetermined			
**Totals**	0	5	0
**TOTAL**			5

**Tallur *et al* (2000)**			
**Organism Isolated**	≤7 days of life	8-59 days of life	60-90 days of life
		0-28	
Staphylococcus aureus		3	
Coagulase Negative Staphylococci		5	
Group A Streptococci/ *Streptococcus Pyogenes*			
Group B Streptococci			
Group D Streptococci/ *Enterococcus*			
Group G Streptococci			
Streptococcus pneumoniae			
Other/unspecified *Streptococcus* species			
Other/ unspecified Gram positives			
**All Gram positives**	0	8	0
Klebsiella pneumoniae		24	
Other/unspecified *Klebsiella* species		4	
Escherichia coli		8	
Pseudomonas species		6	
Enterobacter species			
Serratia species			
Proteus species			
Salmonella species			
Citrobacter species			
Haemophilus influenzae			
Neisseria meningitidis			
Acinetobacter species			
Moraxella species			
Other/unspecified Gram negatives		10	
**All Gram negatives**	0	52	0
Non-stated/Undetermined		2	
**Totals**	0	62	0
**TOTAL**			62

Taskin *et al* (2004)			
**Organism Isolated**	≤7 days of life	8-59 days of life	60-90 days of life
	Early-onset	*Late-onset*	
Staphylococcus aureus	11	15	
Coagulase Negative Staphylococci	4	5	
Group A Streptococci/ *Streptococcus Pyogenes*			
Group B Streptococci			
Group D Streptococci/ *Enterococcus*	1	4	
Group G Streptococci			
Streptococcus pneumoniae			
Other/unspecified *Streptococcus* species			
Other/ unspecified Gram positives			
**All Gram positives**	16	24	0
Klebsiella pneumoniae	1	5	
Other/unspecified *Klebsiella* species			
Escherichia coli	7	11	
Pseudomonas species		4	
Enterobacter species			
Serratia species			
Proteus species			
Salmonella species			
Citrobacter species			
Haemophilus influenzae			
Neisseria meningitidis			
Acinetobacter species			
Moraxella species			
Other/unspecified Gram negatives			
**All Gram negatives**	8	20	0
Non-stated/Undetermined	2	1	
**Totals**	26	45	0
**TOTAL**			71

**Weiss *et al* (2001)**			
**Organism Isolated**	≤7 days of life	8-59 days of life	60-90 days of life
		≤ 2 Months	
Staphylococcus aureus			
Coagulase Negative Staphylococci			
Group A Streptococci/ Streptococcus Pyogenes			
Group B Streptococci			
Group D Streptococci/ *Enterococcus*			
Group G Streptococci			
Streptococcus pneumoniae		1	
Other/unspecified *Streptococcus* species			
Other/ unspecified Gram positives			
**All Gram positives**	0	1	0
Klebsiella pneumoniae			
Other/unspecified *Klebsiella* species			
Escherichia coli			
Pseudomonas species			
Enterobacter species			
Serratia species			
Proteus species			
Salmonella species			
Citrobacter species			
Haemophilus influenzae		3	
Neisseria meningitidis		2	
Acinetobacter species			
Moraxella species			
Other/unspecified Gram negatives			
**All Gram negatives**	0	5	0
Non-stated/Undetermined		27	
**Totals**	0	33	0
**TOTAL**			33

**Yu *et al* (2001)**			
**Organism Isolated**	≤7 days of life	8-59 days of life	60-90 days of life
		Neonates/Newborns	
Staphylococcus aureus		136	
Coagulase Negative Staphylococci		797	
Group A Streptococci/ *Streptococcus Pyogenes*			
Group B Streptococci			
Group D Streptococci/ *Enterococcus*			
Group G Streptococci			
Streptococcus pneumoniae			
Other/unspecified *Streptococcus* species		40	
Other/ unspecified Gram positives		110	
**All Gram positives**	0	1083	0
Klebsiella pneumoniae		130	
Other/unspecified *Klebsiella* species			
Escherichia coli		230	
Pseudomonas species		130	
Enterobacter species		43	
Serratia species		39	
Proteus species			
Salmonella species			
Citrobacter species			
Haemophilus influenzae			
Neisseria meningitidis			
Acinetobacter species		92	
Moraxella species			
Other/unspecified Gram negatives		18	
**All Gram negatives**	0	682	0
Non-stated/Undetermined			
**Totals**	0	1765	0
**TOTAL**			1765
