# Supplementary material for: Aetiology of community-acquired neonatal sepsis in low and middle income countries
Source: J Glob Health. 2011 Dec;1(2):154–70. (PMC3484773)
Supplement: Supplementary Table 3 [file jogh-01-154-s003.pdf]

Supplementary Table 3. Region-specific age-of-onset data for potential pathogens

**Potential pathogens by age of onset and region**	**Africa**			**Americas**			**Eastern Mediterranean**		
Organism Isolated	≤7 days of life	8-59 days of life	60-90 days of life	≤7 days of life	8-59 days of life	60-90 days of life	≤7 days of life	8-59 days of life	60-90 days of life
Staphylococcus aureus	9	76	1	0	0	0	0	0	0
Group A Streptococci/ Streptococcus pyogenes	3	37	1	0	0	0	0	0	0
Group B Streptococci	17	23	0	0	0	0	0	0	0
Group D Streptococci/ Enterococcus	3	1	0	0	0	0	0	2	0
Streptococcus pneumoniae	8	73	7	0	1	0	3	0	0
Other/unspecified Streptococcus species	11	8	0	0	0	0	10	0	0
**Potentially pathogenic Gram positives**	**51**	**218**	**9**	**0**	**1**	**0**	**13**	**2**	**0**
Klebsiella species	23	19	1	0	0	0	0	2	0
Escherichia coli	34	30	1	0	0	0	0	3	0
Pseudomonas species	11	11	1	0	0	0	3	1	0
Enterobacter species	2	3	0	0	0	0	0	4	0
Serratia species	0	0	1	0	0	0	0	0	0
Proteus species	5	4	0	0	0	0	0	0	0
Salmonella species	1	16	4	0	0	0	0	0	0
Haemophilus influenzae	1	24	2	0	3	0	1	0	0
Neisseria meningitidis	0	11	0	0	2	0	0	0	0
Acinetobacter species	16	10	0	0	0	0	0	2	0
**Potentially pathogenic Gram negatives**	**93**	**128**	**10**	**0**	**5**	**0**	**4**	**12**	**0**
Totals	144	346	19	0	6	0	17	14	0
TOTAL			509			6			31

Potential athogens by age of onset and region	Europe			South-East Asia			Western Pacific		
Organism Isolated	≤7 days of life	8-59 days of life	60-90 days of life	≤7 days of life	8-59 days of life	60-90 days of life	≤7 days of life	8-59 days of life	60-90 days of life
Staphylococcus aureus	11	17	0	4	16	0	9	159	0
Group A Streptococci/ Streptococcus pyogenes	0	0	0	0	2	0	1	11	7
Group B Streptococci	0	1	0	1	5	0	1	2	0
Group D Streptococci/ Enterococcus	1	8	0	0	0	0	0	2	0
Streptococcus pneumoniae	0	0	0	2	5	0	0	14	7
Other/unspecified Streptococcus species	0	0	0	1	0	0	2	42	0
**Potentially pathogenic Gram positives**	**12**	**26**	**0**	**8**	**28**	**0**	**13**	**230**	**14**
Klebsiella species	1	19	0	0	72	0	8	135	1
Escherichia coli	7	31	0	1	17	0	4	239	0
Pseudomonas species	0	4	0	2	16	0	6	134	0
Enterobacter species	0	1	0	1	2	0	7	49	0
Serratia species	0	1	0	0	0	0	0	39	0
Proteus species	0	1	0	0	0	0	1	2	0
Salmonella species	0	0	0	0	3	0	0	7	2
Haemophilus influenzae	0	0	0	0	1	0	0	2	2
Neisseria meningitidis	0	0	0	0	0	0	0	0	0
Acinetobacter species	0	0	0	2	7	0	1	94	3
**Potentially pathogenic Gram negatives**	**8**	**57**	**0**	**6**	**118**	**0**	**27**	**701**	**8**
Totals	20	83	0	14	146	0	40	931	22
TOTAL			103			160			993
