# Supplementary material for: Aetiology of community-acquired neonatal sepsis in low and middle income countries
Source: J Glob Health. 2011 Dec;1(2):154–70. (PMC3484773)
Supplement: Supplementary Table 4 [file jogh-01-154-s004.pdf]

Supplementary Table 4. Regional tables for all age-of-onset categories

**All organisms isolated by region**	**Africa**		**Americas**		**South-East Asia**		**Europe**		**Eastern Mediterranean**		**Western Pacific**		**All Regions**	
**Organism Isolated**	N	%	N	%	N	%	N	%	N	%	N	%	N	%
Staphylococcus aureus	86	14.2	0	0.0	20	10.0	28	26.4	0	0.0	168	14.9	302	14.3
Group A Streptococci/ Streptococcus pyogenes	41	6.8	0	0.0	2	1.0	0	0.0	0	0.0	19	1.7	62	2.9
Group B Streptococci	40	6.6	0	0.0	6	3.0	1	0.9	0	0.0	3	0.3	50	2.4
Group D Streptococci/ Enterococcus	4	0.7	0	0.0	0	0.0	9	8.5	2	4.8	2	0.2	17	0.8
Streptococcus pneumoniae	88	14.6	1	3.0	7	3.5	0	0.0	3	7.1	21	1.9	120	5.7
Other/unspecified Streptococcus species	19	3.1	0	0.0	1	0.5	0	0.0	10	23.8	44	3.9	74	3.5
Other/ unspecified Gram positives	1	0.2	0	0.0	1	0.5	0	0.0	0	0.0	110	9.8	112	5.3
**All Gram positives**	**279**	**46.2**	**1**	**3.0**	**37**	**18.5**	**38**	**35.8**	**15**	**35.7**	**367**	**32.6**	**737**	**34.9**
Klebsiella pneumoniae	23	3.8	0	0.0	67	33.5	20	18.9	2	4.8	144	12.8	256	12.1
Other/unspecified Klebsiella species	20	3.3	0	0.0	5	2.5	0	0.0	0	0.0	0	0.0	25	1.2
Escherichia coli	65	10.8	0	0.0	18	9.0	38	35.8	3	7.1	243	21.6	367	17.4
Pseudomonas species	23	3.8	0	0.0	18	9.0	4	3.8	4	9.5	140	12.4	189	9.0
Enterobacter species	5	0.8	0	0.0	3	1.5	1	0.9	4	9.5	56	5.0	69	3.3
Serratia species	1	0.2	0	0.0	0	0.0	1	0.9	0	0.0	39	3.5	41	1.9
Proteus species	9	1.5	0	0.0	0	0.0	1	0.9	0	0.0	3	0.3	13	0.6
Salmonella species	21	3.5	0	0.0	3	1.5	0	0.0	0	0.0	9	0.8	33	1.6
Haemophilus influenzae	27	4.5	3	9.1	1	0.5	0	0.0	1	2.4	4	0.4	36	1.7
Neisseria meningitidis	11	1.8	2	6.1	0	0.0	0	0.0	0	0.0	0	0.0	13	0.6
Acinetobacter species	26	4.3	0	0.0	9	4.5	0	0.0	2	4.8	98	8.7	135	6.4
Other/unspecified Gram negatives	59	9.8	0	0.0	11	5.5	0	0.0	11	26.2	21	1.9	102	4.8
**All Gram negatives**	**290**	**48.0**	**5**	**15.2**	**135**	**67.5**	**65**	**61.3**	**27**	**64.3**	**757**	**67.3**	**1279**	**60.6**
Non-stated/Undetermined	35	5.8	27	81.8	28	14.0	3	2.8	0	0.0	1	0.1	94	4.5
**Total**	**604**	**100.0**	**33**	**100.0**	**200**	**100.0**	**106**	**100.0**	**42**	**100.0**	**1125**	**100.0**	**2110**	**100.0**

Data was also extracted for Coagulase negative Staphylococci: Africa – 28 isolates, Americas – 0 isolates, South-East Asia – 35 isolates, Europe – 9 isolates, Eastern Mediterranean – 0 isolates, Western Pacific – 811 isolates

Potential Pathogens by Region	Africa		Americas		Eastern Mediterranean		Europe		South-East Asia		Western Pacific		All Regions	
**Organism Isolated**	N	%	N	%	N	%	N	%	N	%	N	%	N	%
Staphylococcus aureus	86	16.9	0	0.0	0	0.0	28	27.2	20	12.5	168	16.9	302	16.8
Group A Streptococci/ Streptococcus pyogenes	41	8.1	0	0.0	0	0.0	0	0.0	2	1.3	19	1.9	62	3.4
Group B Streptococci	40	7.9	0	0.0	0	0.0	1	1.0	6	3.8	3	0.3	50	2.8
Group D Streptococci/ Enterococcus	4	0.8	0	0.0	2	6.5	9	8.7	0	0.0	2	0.2	17	0.9
Streptococcus pneumoniae	88	17.3	1	16.7	3	9.7	0	0.0	7	4.4	21	2.1	120	6.7
Other/unspecified Streptococcus species	19	3.7	0	0.0	10	32.3	0	0.0	1	0.6	44	4.4	74	4.1
**Potentially pathogenic Gram positives**	**278**	**54.6**	**1**	**16.7**	**15**	**48.4**	**38**	**36.9**	**36**	**22.5**	**257**	**25.9**	**625**	**34.7**
Klebsiella species	43	8.4	0	0.0	2	6.5	20	19.4	72	45.0	144	14.5	281	15.6
Escherichia coli	65	12.8	0	0.0	3	9.7	38	36.9	18	11.3	243	24.5	367	20.4
Pseudomonas species	23	4.5	0	0.0	4	12.9	4	3.9	18	11.3	140	14.1	189	10.5
Enterobacter species	5	1.0	0	0.0	4	12.9	1	1.0	3	1.9	56	5.6	69	3.8
Serratia species	1	0.2	0	0.0	0	0.0	1	1.0	0	0.0	39	3.9	41	2.3
Proteus species	9	1.8	0	0.0	0	0.0	1	1.0	0	0.0	3	0.3	13	0.7
Salmonella species	21	4.1	0	0.0	0	0.0	0	0.0	3	1.9	9	0.9	33	1.8
Haemophilus influenzae	27	5.3	3	50.0	1	3.2	0	0.0	1	0.6	4	0.4	36	2.0
Neisseria meningitidis	11	2.2	2	33.3	0	0.0	0	0.0	0	0.0	0	0.0	13	0.7
Acinetobacter species	26	5.1	0	0.0	2	6.5	0	0.0	9	5.6	98	9.9	135	7.5
**Potentially pathogenic Gram negatives**	**231**	**45.4**	**5**	**83.3**	**16**	**51.6**	**65**	**63.1**	**124**	**77.5**	**736**	**74.1**	**1177**	**65.3**
**Total**	**509**	**100.0**	**6**	**100.0**	**31**	**100.0**	**103**	**100.0**	**160**	**100.0**	**993**	**100.0**	**1802**	**100.0**
