# Supplementary material for: Aetiology of community-acquired neonatal sepsis in low and middle income countries
Source: J Glob Health. 2011 Dec;1(2):154–70. (PMC3484773)
Supplement: Supplementary Table 5 [file jogh-01-154-s005.pdf]

Supplementary Table 5. Meta-analysis forest plot graphs

Staphylococcus aureus

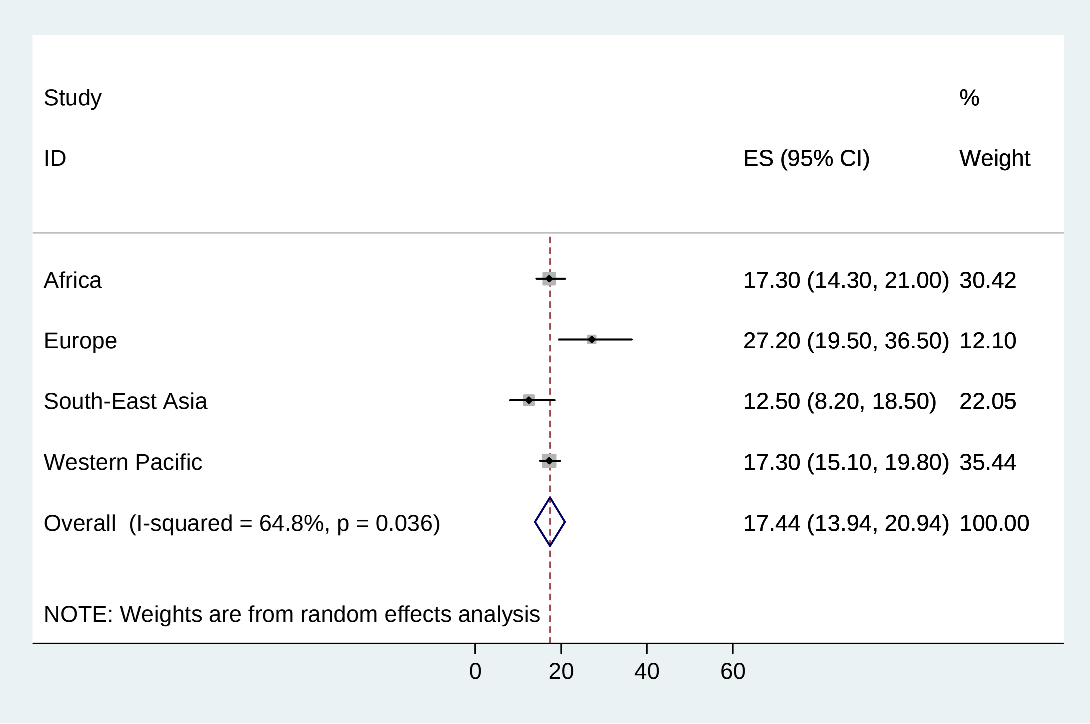

Group A *Streptococci/ Streptococcus pyogenes*

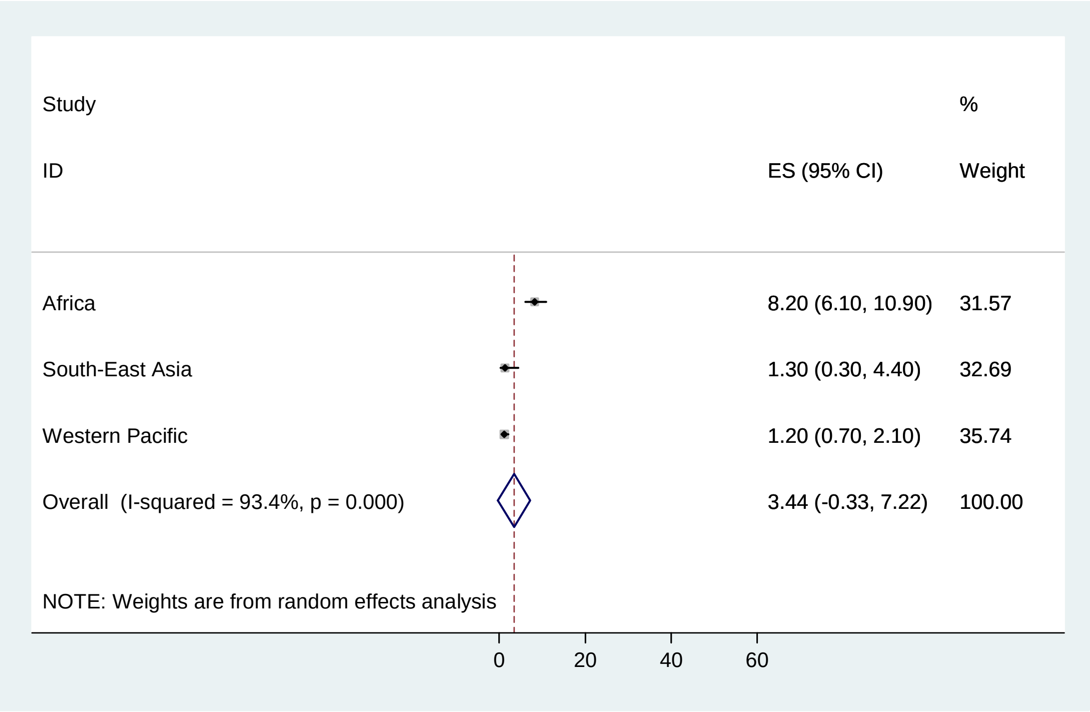

Group B *Streptococci*

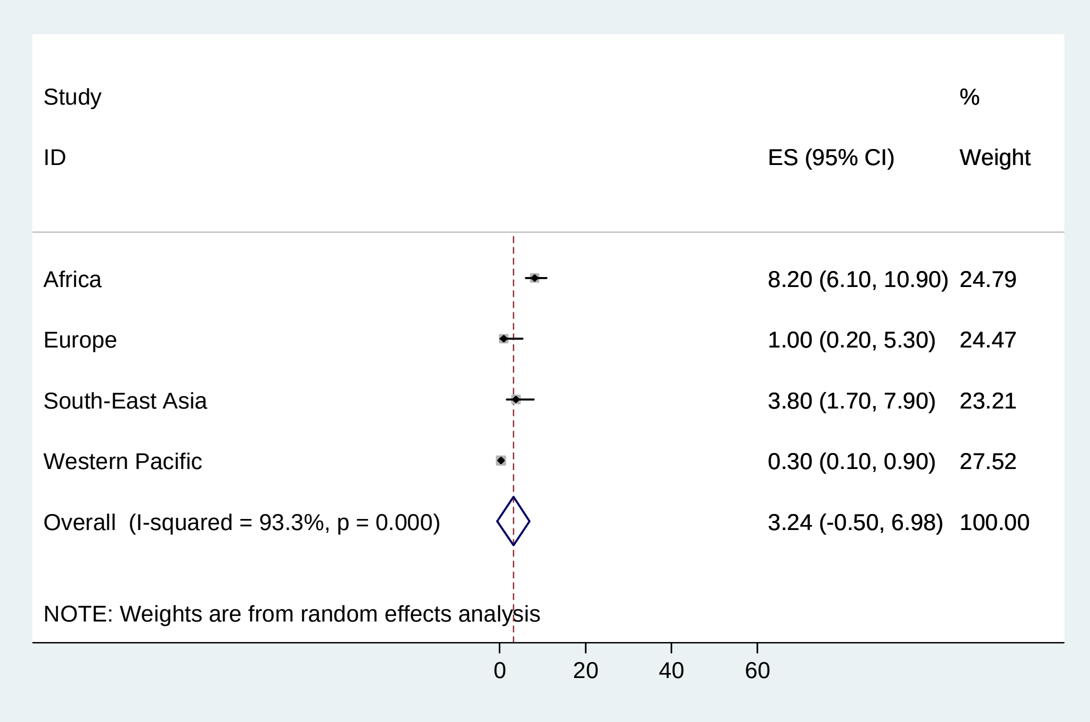

Group D *Streptococci/ Enterococcus*

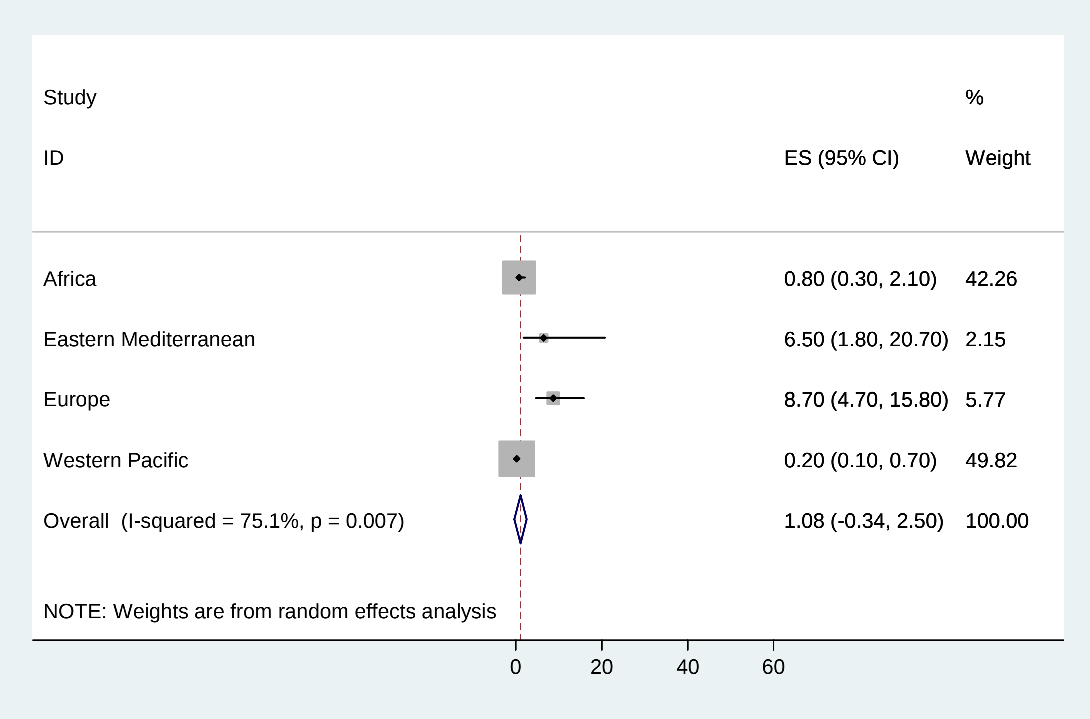

Streptococcus pneumoniae

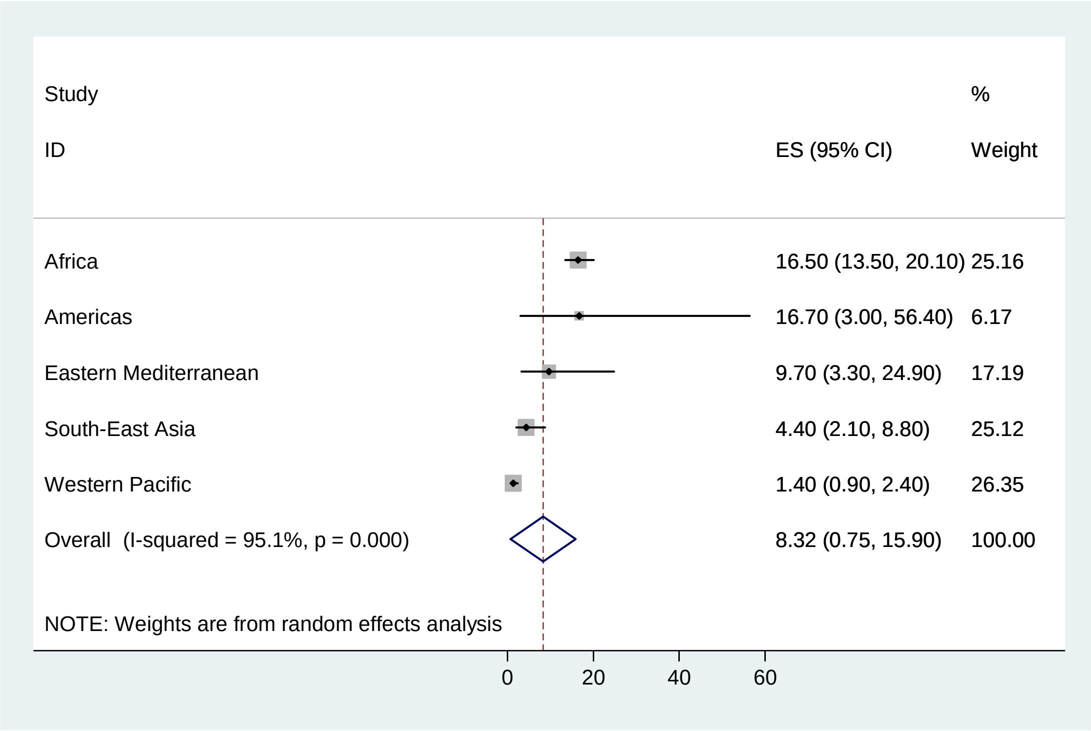

Other/Unspecified *Streptococcus* species

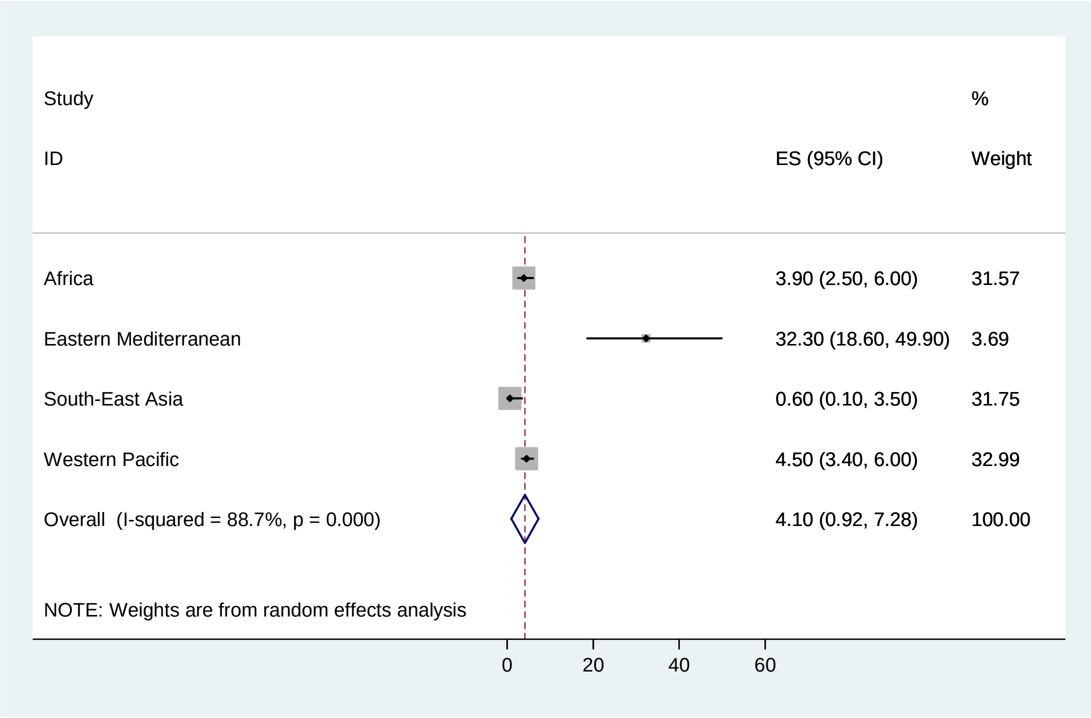

Klebsiella species

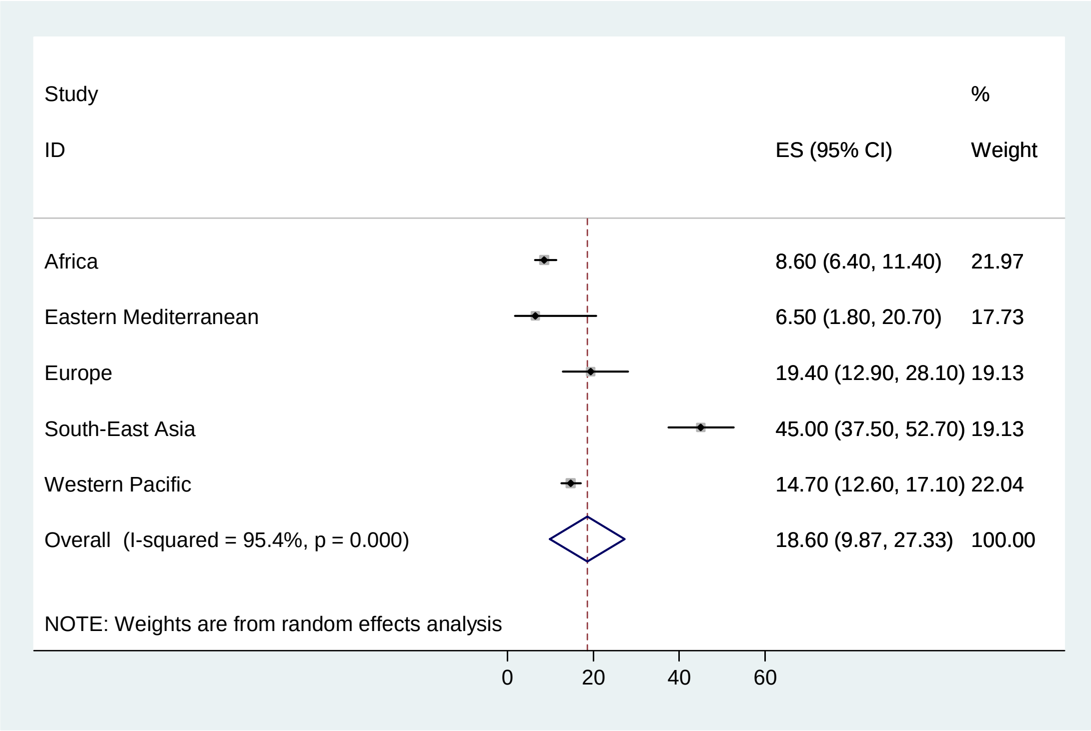

Escherichia coli

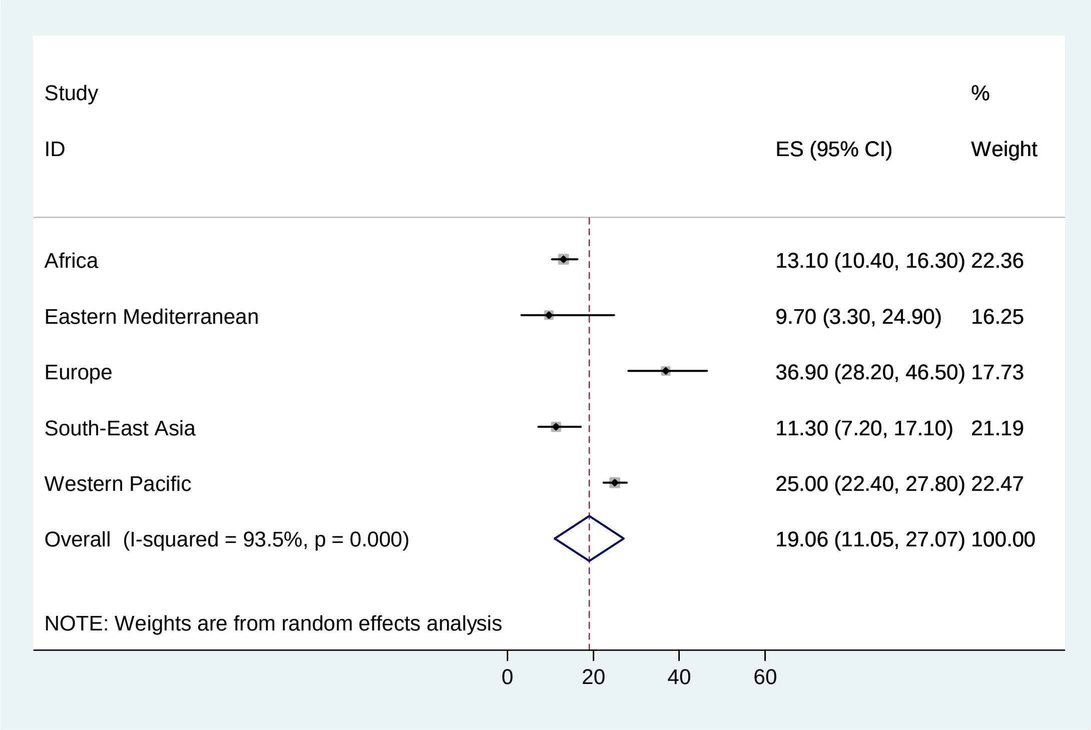

Pseudomonas species

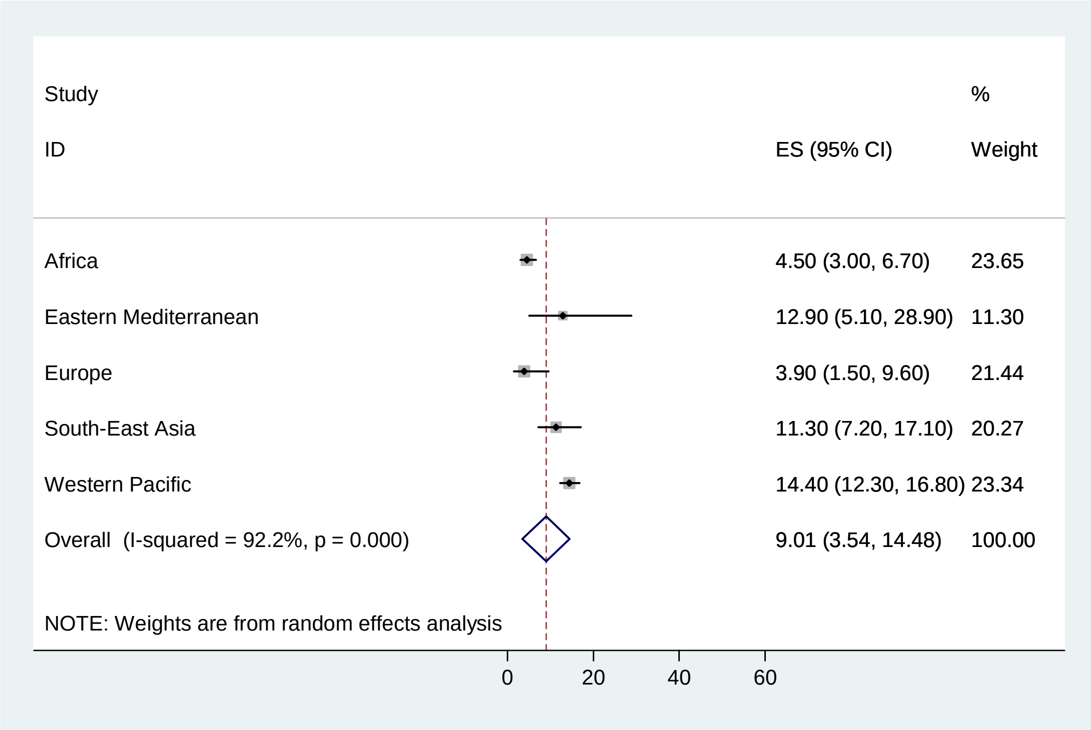

Enterobacter species

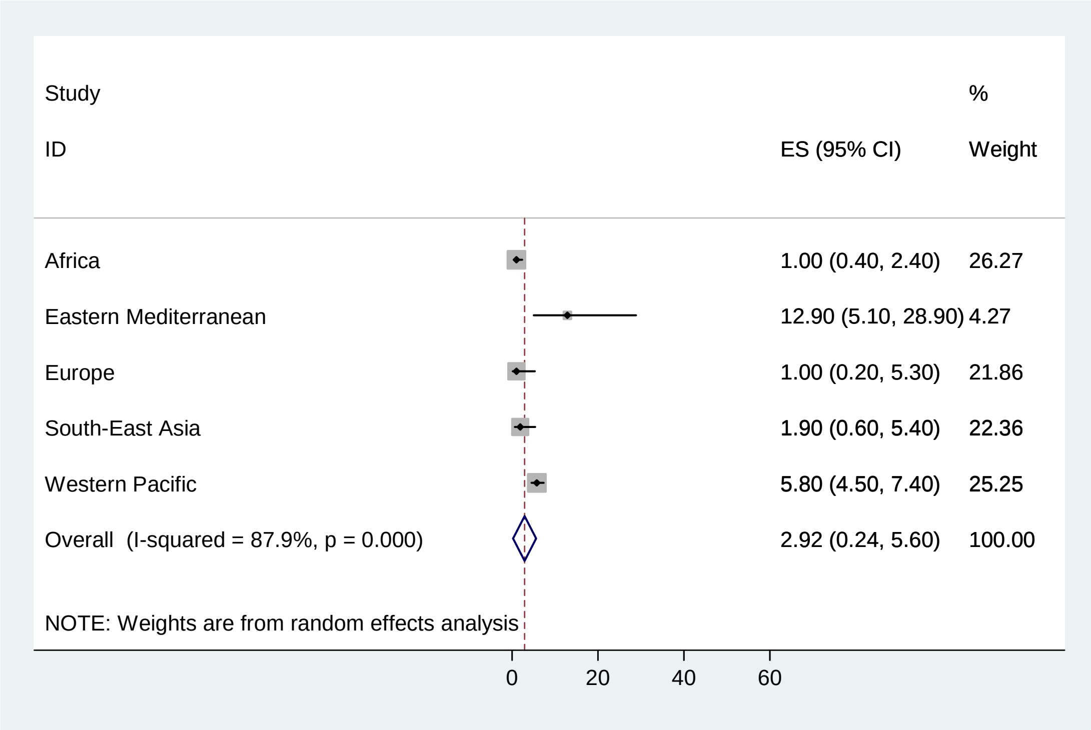

Proteus species

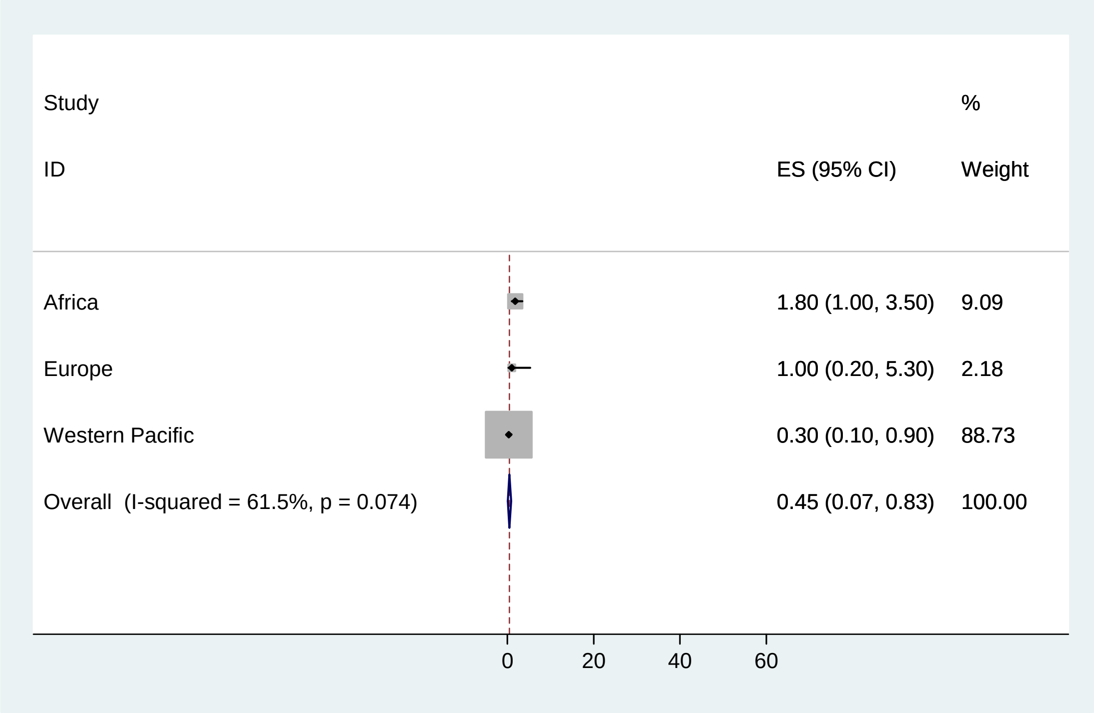

Salmonella species

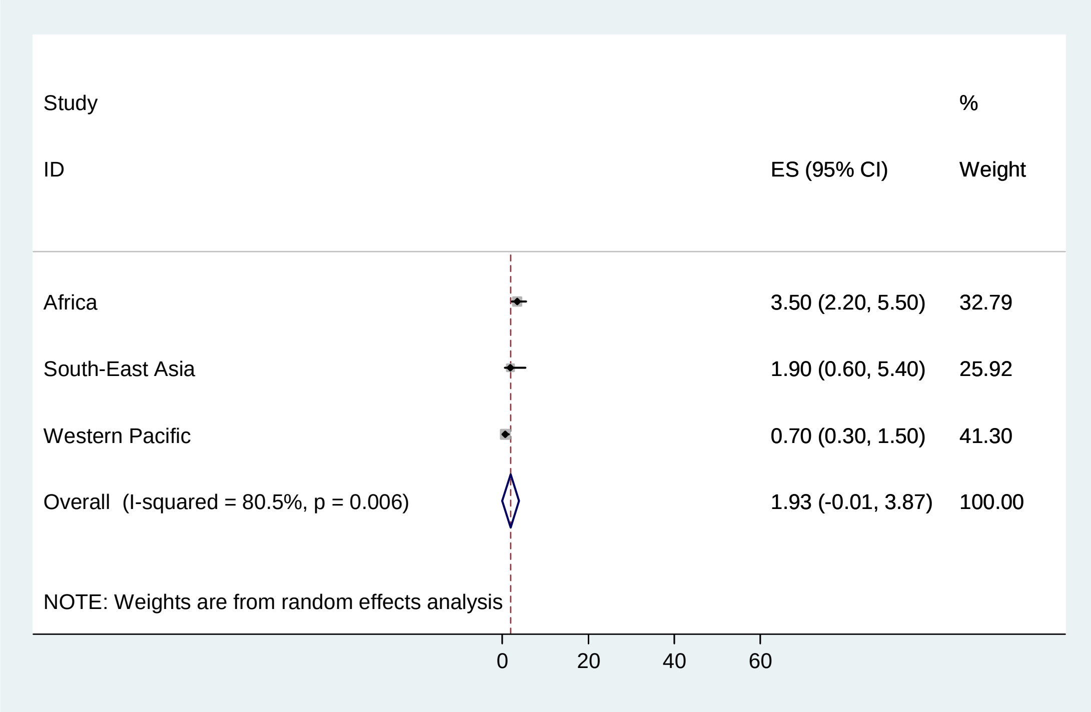

Haemophilus influenzae

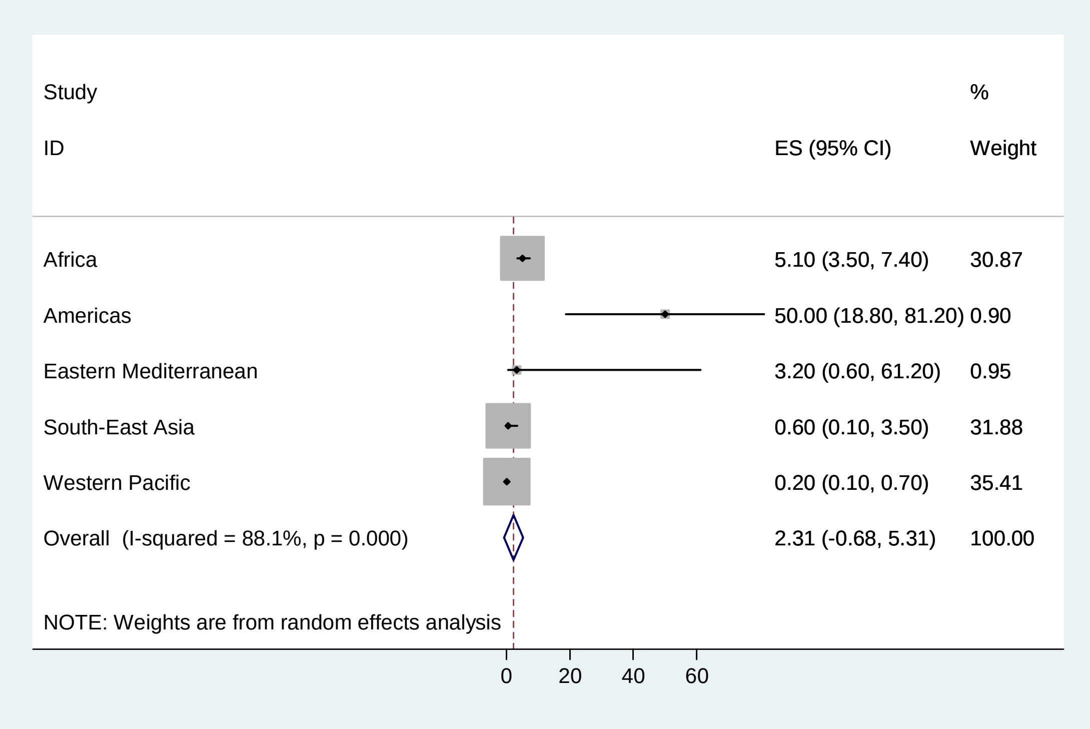

Acinetobacter species

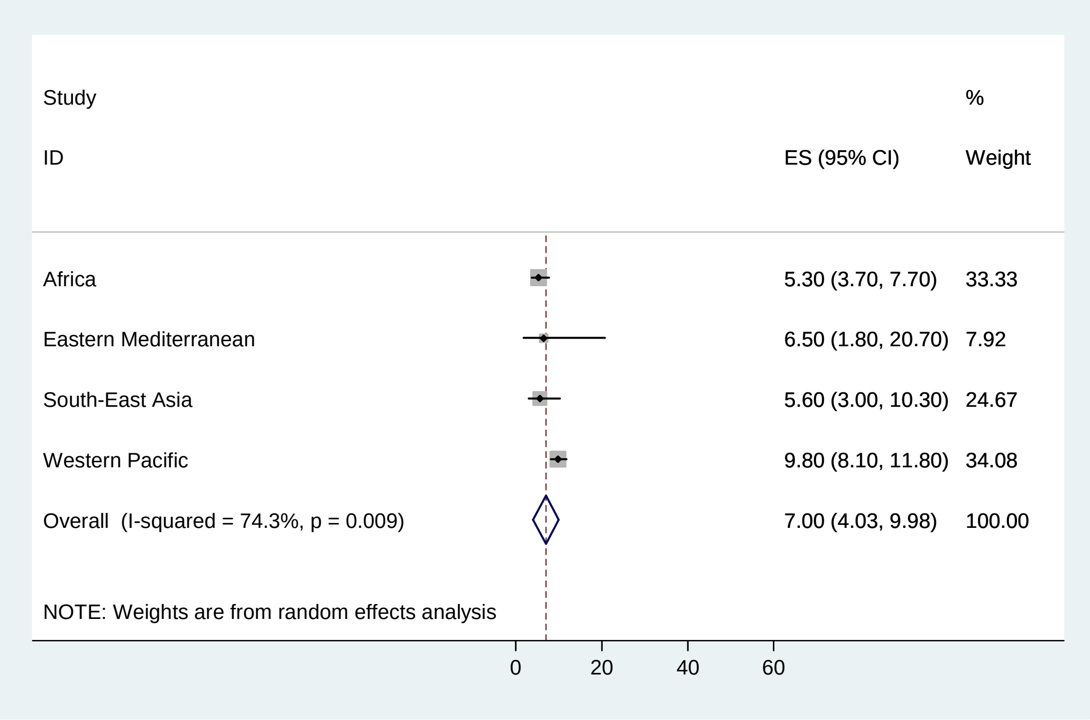

Forest plots not possible for *Serratia* species or *Neisseria meningitidis* due to small numbers of studies.
