# Supplementary material for: Aetiology of community-acquired neonatal sepsis in low and middle income countries
Source: J Glob Health. 2011 Dec;1(2):154–70. (PMC3484773)
Supplement: Supplementary Table 6 [file jogh-01-154-s006.pdf]

Supplementary Table 6. Inconclusive studies – individual and overall tables

**Adhikari *et al* (1995)**			
**Organism Isolated**	≤7 days of life	8-59 days of life	60-90 days of life
Staphylococcus aureus	0	0	
Coagulase Negative Staphylococci			
Group A Streptococci/ *Streptococcus Pyogenes*			
Group B Streptococci	14	13	
Group D Streptococci/ *Enterococcus*			
Group G Streptococci			
Streptococcus pneumoniae	1	3	
Other/unspecified *Streptococcus* species			
Other/ unspecified Gram positives			
**All Gram positives**	15	16	0
Klebsiella pneumoniae	11	8	
Other/unspecified *Klebsiella* species			
Escherichia Coli	7	5	
Pseudomonas species	1	1	
Enterobacter species		1	
Serratia species			
Proteus species		2	
Salmonella species		2	
Citrobacter species			
Haemophilus influenzae			
Nisseria meningitidis			
Acinetobacter species		1	
Moraxella species			
Other/unspecified Gram negatives			
**All Gram negatives**	19	20	0
Non-stated/Undetermined			
**Totals**	34	36	0
**TOTAL**			70

**Al-Zwaini *et al* (2002)**			
**Organism Isolated**	≤7 days of life	8-59 days of life	60-90 days of life
		0-28 days	
Staphylococcus aureus		22	
Coagulase Negative Staphylococci			
Group A Streptococci/ *Streptococcus Pyogenes*			
Group B Streptococci		0	
Group D Streptococci/ *Enterococcus*			
Group G Streptococci			
Streptococcus pneumoniae		1	
Other/unspecified *Streptococcus* species			
Other/ unspecified Gram positives			
**All Gram positives**	0	23	0
Klebsiella pneumoniae		4	
Other/unspecified *Klebsiella* species			
Escherichia Coli		5	
Pseudomonas species		2	
Enterobacter species			
Serratia species		2	
Proteus species		2	
Salmonella species		1	
Citrobacter species			
Haemophilus influenzae			
Nisseria meningitidis		1	
Acinetobacter species			
Moraxella species			
Other/unspecified Gram negatives			
**All Gram negatives**	0	17	0
Non-stated/Undetermined			
**Totals**	0	40	0
**TOTAL**			40

**Chaturvedi *et al* (1989)**			
**Organism Isolated**	≤7 days of life	8-59 days of life	60-90 days of life
		Neonates/Newborns	
Staphylococcus aureus			
Coagulase Negative Staphylococci (CONS)		254	
Group A Streptococci/ *Streptococcus Pyogenes*			
Group B Streptococci			
Group D Streptococci/ *Enterococcus*			
Group G Streptococci			
Streptococcus pneumoniae			
Other/unspecified *Streptococcus* species		35	
Other/ unspecified Gram positives		126	
**All Gram positives**	0	415	0
Klebsiella pneumoniae			
Other/unspecified *Klebsiella* species		259	
Escherichia Coli		168	
Pseudomonas species		142	
Enterobacter species		1	
Serratia species			
Proteus species		10	
Salmonella species		19	
Citrobacter species		3	
Haemophilus influenzae			
Nisseria meningitidis			
Acinetobacter species		23	
Moraxella species		2	
Other/unspecified Gram negatives		8	
**All Gram negatives**	0	635	0
Non-stated/Undetermined			
**Totals**	0	1050	0
**TOTAL**			1050

**Fadero *et al* (2007)**			
**Organism Isolated**	≤7 days of life	8-59 days of life	60-90 days of life
		Early and late onset	
Staphylococcus aureus		14	
Coagulase Negative Staphylococci		1	
Group A Streptococci/ *Streptococcus Pyogenes*			
Group B Streptococci			
Group D Streptococci/ *Enterococcus*			
Group G Streptococci			
Streptococcus pneumoniae			
Other/unspecified *Streptococcus* species			
Other/ unspecified Gram positives		1	
**All Gram positives**	0	16	0
Klebsiella pneumoniae		1	
Other/unspecified *Klebsiella* species			
Escherichia Coli		2	
Pseudomonas species			
Enterobacter species			
Serratia species			
Proteus species		2	
Salmonella species			
Citrobacter species			
Haemophilus influenzae			
Nisseria meningitidis			
Acinetobacter species			
Moraxella species			
Other/unspecified Gram negatives			
**All Gram negatives**	0	5	0
Non-stated/Undetermined			
**Totals**	0	21	0
**TOTAL**			21

**Milledge *et al* (2005)**			
**Organism Isolated**	≤7 days of life	8-59 days of life	60-90 days of life
		8-30 days	
Staphylococcus aureus	57	30	
Coagulase Negative Staphylococci			
Group A Streptococci/ *Streptococcus Pyogenes*	15	41	
Group B Streptococci	61	75	
Group D Streptococci/ *Enterococcus*			
Group G Streptococci			
Streptococcus pneumoniae	25	54	
Other/unspecified *Streptococcus* species			
Other/ unspecified Gram positives	49	23	
**All Gram positives**	207	223	0
Klebsiella pneumoniae			
Other/unspecified *Klebsiella* species	41	19	
Escherichia Coli	41	26	
Pseudomonas species			
Enterobacter species			
Serratia species			
Proteus species			
Salmonella species	20	90	
Citrobacter species			
Haemophilus influenzae			
Nisseria meningitidis			
Acinetobacter species			
Moraxella species			
Other/unspecified Gram negatives	71	46	
**All Gram negatives**	173	181	0
Non-stated/Undetermined			
**Totals**	380	404	0
**TOTAL**			784

Rao *et al* (1993)			
**Organism Isolated**	≤7 days of life	8-59 days of life	60-90 days of life
		>7 days	
Staphylococcus aureus	32	28	
Coagulase Negative Staphylococci	25	23	
Group A Streptococci/ *Streptococcus Pyogenes*	0	1	
Group B Streptococci			
Group D Streptococci/ *Enterococcus*	0	2	
Group G Streptococci			
Streptococcus pneumoniae			
Other/unspecified *Streptococcus* species			
Other/ unspecified Gram positives			
**All Gram positives**	57	54	0
Klebsiella pneumoniae			
Other/unspecified *Klebsiella* species	18	5	
Escherichia Coli	12	8	
Pseudomonas species	10	20	
Enterobacter species	0	2	
Serratia species			
Proteus species			
Salmonella species	2	13	
Citrobacter species	10	14	
Haemophilus influenzae			
Nisseria meningitidis			
Acinetobacter species	13	10	
Moraxella species			
Other/unspecified Gram negatives	2	5	
**All Gram negatives**	67	77	0
Non-stated/Undetermined			
**Totals**	124	131	0
**TOTAL**			255

**Saxena *et al* (1980)**			
**Organism Isolated**	≤7 days of life	8-59 days of life	60-90 days of life
		Neonates/Newborns	
Staphylococcus aureus		6	
Coagulase Negative Staphylococci			
Group A Streptococci/ *Streptococcus Pyogenes*			
Group B Streptococci			
Group D Streptococci/ *Enterococcus*			
Group G Streptococci			
Streptococcus pneumoniae			
Other/unspecified *Streptococcus* species			
Other/ unspecified Gram positives		8	
**All Gram positives**	0	14	0
Klebsiella pneumoniae			
Other/unspecified *Klebsiella* species		1	
Escherichia Coli			
Pseudomonas species			
Enterobacter species			
Serratia species			
Proteus species			
Salmonella species			
Citrobacter species			
Haemophilus influenzae			
Nisseria meningitidis			
Acinetobacter species			
Moraxella species			
Other/unspecified Gram negatives		12	
**All Gram negatives**	0	13	0
Non-stated/Undetermined			
**Totals**	0	27	0
**TOTAL**			27

Inconclusive studies: all isolates by Age-of-Onset	≤7 days of life		8-59 days of life		60-90 days of life	
**Organism Isolated**	N	%	N	%	N	%
Staphylococcus aureus	89	17.3	100	19.5	0	0.0
Group A Streptococci/ Streptococcus Pyogenes	15	2.9	42	8.2	0	0.0
Group B Streptococci	75	14.6	88	17.2	0	0.0
Group D Streptococci/ Enterococcus	0	0.0	2	0.4	0	0.0
Streptococcus pneumoniae	26	5.1	58	11.3	0	0.0
Other/unspecified Streptococcus species	0	0.0	35	6.8	0	0.0
Other/ unspecified Gram positives	49	9.6	158	30.8	0	0.0
**All Gram positives**	254	49.5	483	94.2	0	0.0
Klebsiella pneumoniae	11	2.1	13	2.5	0	0.0
Other/unspecified Klebsiella species	59	11.5	284	55.4	0	0.0
Escherichia Coli	60	11.7	214	41.7	0	0.0
Pseudomonas species	11	2.1	165	32.2	0	0.0
Enterobacter species	0	0.0	4	0.8	0	0.0
Serratia species	0	0.0	2	0.4	0	0.0
Proteus species	0	0.0	16	3.1	0	0.0
Salmonella species	22	4.3	125	24.4	0	0.0
Haemophilus influenzae	0	0.0	0	0.0	0	0.0
Neisseria meningitidis	0	0.0	1	0.2	0	0.0
Acinetobacter species	13	2.5	34	6.6	0	0.0
Other/unspecified Gram negatives*	83	16.2	90	17.5	0	0.0
**All Gram negatives**	259	50.5	948	184.8	0	0.0
Non-stated/Undetermined	0	0.0	0	0.0	0	0.0
**Totals**	513	100.0	1431	278.9	0	0.0

* Includes data for *Citrobacter* Spp., *Moraxella* Spp., *Shigella* Spp., *Aeromonas* Spp. and others

Also extracted data for *Coagulase Negative Staphylococci*: ≤ 7 days of life – 25, 8-59 days of life – 278, 60-90 days of life – 0

Inconclusive studies: All organisms isolated		
**Organism Isolated**	**N**	**%**
Staphylococcus aureus	189	9.7
Group A Streptococci/ *Streptococcus Pyogenes*	57	2.9
Group B Streptococci	163	8.4
Group D Streptococci/ *Enterococcus*	2	0.1
Streptococcus pneumoniae	84	4.3
Other/unspecified *Streptococcus* species	35	1.8
Other/ unspecified Gram positives	207	10.6
**All Gram positives**	**737**	**37.9**
Klebsiella pneumoniae	24	1.2
Other/unspecified *Klebsiella* species	343	17.6
Escherichia Coli	274	14.1
Pseudomonas species	176	9.1
Enterobacter species	4	0.2
Serratia species	2	0.1
Proteus species	16	0.8
Salmonella species	147	7.6
Haemophilus influenzae	0	0.0
Neisseria meningitidis	1	0.1
Acinetobacter species	47	2.4
Other/unspecified Gram negatives*	173	8.9
**All Gram negatives**	**1207**	**62.1**
Non-stated/Undetermined	0	0.0
**Total**	**1944**	**100.0**

* Includes data for *Citrobacter* Spp., *Moraxella* Spp., *Shigella* Spp., Aeromonas Spp. and others

Also extracted data for *Coagulase Negative Staphylococci:* 303 cases

Inconclusive Studies: All Potential Pathogens Isolated		
**Organism Isolated**	**N**	**%**
Staphylococcus aureus	189	12.1
Group A Streptococci/ Streptococcus Pyogenes	57	3.6
Group B Streptococci	163	10.4
Group D Streptococci/ Enterococcus	2	0.1
Streptococcus pneumoniae	84	5.4
Other/unspecified Streptococcus species	35	2.2
**Potentially Pathogenic Gram positives**	**530**	**33.9**
Klebsiella pneumoniae	24	1.5
Other/unspecified Klebsiella species	343	21.9
Escherichia Coli	274	17.5
Pseudomonas species	176	11.3
Enterobacter species	4	0.3
Serratia species	2	0.1
Proteus species	16	1.0
Salmonella species	147	9.4
Haemophilus influenzae	0	0.0
Neisseria meningitidis	1	0.1
Acinetobacter species	47	3.0
**Potentially Pathogenic Gram negatives**	**1034**	**66.1**
**Total**	**1564**	**100.0**
